# Small molecule modulation of splicing factor expression is associated with rescue from cellular senescence

**DOI:** 10.1186/s12860-017-0147-7

**Published:** 2017-10-17

**Authors:** Eva Latorre, Vishal C. Birar, Angela N. Sheerin, J. Charles C. Jeynes, Amy Hooper, Helen R. Dawe, David Melzer, Lynne S. Cox, Richard G. A. Faragher, Elizabeth L. Ostler, Lorna W. Harries

**Affiliations:** 1 Institute of Biomedical and Clinical Sciences, University of Exeter Medical School, University of Exeter, Barrack Road, Exeter, Devon EX2 5DW UK; 2 School of Pharmacy and Biomolecular Sciences, University of Brighton, Cockcroft Building, Moulsecoomb, Brighton, BN2 4GJ UK; 3 Centre for Biomedical Modelling and Analysis, University of Exeter, Exeter, Devon EX2 5DW UK; 4 College of Life and Environmental Sciences, University of Exeter, Exeter, Devon EX4 4QD UK; 5 Department of Biochemistry, University of Oxford, Oxford, OX1 3QU UK

**Keywords:** Alternative splicing, Ageing, Resveratrol, Senescence, Fibroblasts

## Abstract

**Background:**

Altered expression of mRNA splicing factors occurs with ageing in vivo and is thought to be an ageing mechanism. The accumulation of senescent cells also occurs in vivo with advancing age and causes much degenerative age-related pathology. However, the relationship between these two processes is opaque. Accordingly we developed a novel panel of small molecules based on resveratrol, previously suggested to alter mRNA splicing, to determine whether altered splicing factor expression had potential to influence features of replicative senescence.

**Results:**

Treatment with resveralogues was associated with altered splicing factor expression and rescue of multiple features of senescence. This rescue was independent of cell cycle traverse and also independent of SIRT1, SASP modulation or senolysis. Under growth permissive conditions, cells demonstrating restored splicing factor expression also demonstrated increased telomere length, re-entered cell cycle and resumed proliferation. These phenomena were also influenced by ERK antagonists and agonists.

**Conclusions:**

This is the first demonstration that moderation of splicing factor levels is associated with reversal of cellular senescence in human primary fibroblasts. Small molecule modulators of such targets may therefore represent promising novel anti-degenerative therapies.

**Electronic supplementary material:**

The online version of this article (10.1186/s12860-017-0147-7) contains supplementary material, which is available to authorized users.

## Background

Messenger RNA (mRNA) processing has been implicated as a key determinant of lifespan. Splicing factor expression is dysregulated in the peripheral blood of aging humans, where they are the major functional gene ontology class whose transcript patterns alter with advancing age [1] and in senescent primary human cells of multiple lineages [2]. Splicing factor expression is also an early determinant of longevity in mouse and man [3], and in both species these changes are likely to be functional, since they are associated with alterations in splice site usage for many genes [1–3]. Recent data suggests that modification of the levels of SFA-1, a core component of the spliceosome, influences lifespan in *C. elegans* through interaction with TORC1 machinery [4]. Diseases for which age is a significant risk factor including Alzheimer’s disease [5], Parkinson’s disease [6] and cancer [7] are also marked by major changes in the isoform repertoires, highlighting the importance of correct splicing for health throughout the life course. Thus, the loss of fine-tuning of gene expression in ageing tissues and the resulting failure to respond appropriately to intrinsic and extrinsic cellular stressors has the potential to be a major contributor to the increased physiological frailty seen in aging organisms [8].

The splicing process is regulated on two levels. Firstly, constitutive splicing is carried out by the core spliceosome, which recognises splice donor and acceptor sites that define introns and exons. However, fine control of splice site usage is orchestrated by a complex interplay between splicing regulator proteins such as the Serine Arginine (SR) class of splicing activators and the heterogeneous ribonucleoprotein (hnRNP) class of splicing repressors. Splicing activators bind to exon and intron splicing enhancers (ESE, ISE), and splicing inhibitors to intron and exon splicing silencers (ESS, ISS). Splice site usage relies on the balance between these factors and occurs in a concentration-dependent manner [9–11]. Other aspects of information transfer from DNA to protein, such as RNA export and mRNA stability are also influenced by splicing factors [12]. Intriguingly, in addition to their splicing roles, many splicing factors have non-canonical additional functions regulating processes relevant to ageing. For example, hnRNPK, hnRNPD and hnRNPA1 have been shown to have roles in telomere maintenance [13–15], hnRNPA1 regulates the stability of SIRT1 mRNA transcripts [16] and hnRNPA2/B1 is involved in maintenance of stem cell populations [17]. Splicing factor expression is known to be dysregulated in senescent cells of multiple lineages [2] and it is now well established that the accumulation of senescent cells is a direct cause of multiple aspects of both ageing and age-related disease in mammals [18].

Senescent cells accumulate progressively through life in a variety of mammalian species [15], and premature senescence is a hallmark of many human progeroid syndromes. Conversely, dietary restriction, which increases longevity, retards the accumulation of senescent cells. Most compellingly, deletion of senescent cells in transgenic mice improves multiple aspects of later life health and extends lifespan [19]. The mechanisms by which senescent cells mediate these deleterious effects are complex but include factors such as ectopic calcification in the case of vascular smooth muscle cells [20] and secretion of pro-inflammatory cytokines, the well-known Senescence Associated Secretory Phenotype (SASP) [21]. These observations suggest that an interrelationship may exist between well characterised mechanisms of ageing, such as cellular senescence, and the RNA splicing machinery where the mechanistic relationship to ageing remains largely correlational.

In contrast to the situation with core spliceosomal proteins such as SFA-1, perturbation of a single splicing regulator by standard molecular techniques such as knockdown or overexpression is unlikely to be informative for assessment of effects on ageing and cell senescence, since ageing is characterised by co-ordinate dysregulation of large modules of splicing factors [1, 2]. Splice site choice is also dependent on the balance between more than a hundred splicing activator and splicing inhibitor regulatory proteins, which differ from splice site to splice site and from tissue to tissue [9, 10]. Thus experimental tools capable of co-ordinately modulating the expression of multiple components simultaneously are required to address the potential effects of the dysregulation of large numbers of splicing factors that we note during the ageing process. Small molecules such as resveratrol have been reported to influence splicing regulatory factor expression in transformed cell lines such as HEK293 and HeLa [22], although it is not yet known whether this is a direct or indirect effect. Unfortunately, resveratrol has multiple biological effects, including a reduction of pro-inflammatory cytokine expression [23] as well as its canonical activity against SIRT1 [24] thus a ‘clean’ assessment of the effects of moderation of splicing factor levels on cell physiology cannot be achieved using this compound alone.

We have overcome this limitation through development of a novel library of resveratrol-related compounds (resveralogues) which are all capable of either directly or indirectly influencing the expression of multiple splicing factors of both *SRSF* and *HNRNP* subtypes, whilst exhibiting differential activity against SIRT1 and SASP. Treatment of senescent human fibroblasts from different developmental lineages with any of these novel molecules shifts expression patterns of multiple splicing factors to those characteristic of much earlier passage cells. This change occurs regardless of cell cycle traverse and is associated with a marked decrease in key biochemical and molecular biomarkers of senescence without any significant alteration in levels of apoptosis. Elevated splicing factor expression is also associated with elongation of telomeres, and in growth permissive conditions, these previously senescent populations show significant increases in growth fraction (as measured by Ki67 staining) and in absolute cell number, indicating cell cycle re-entry. The mechanisms by which ‘rejuvenation’ occurs are independent both of SIRT1 activation, or effects on the SASP. Thus, molecules that modulate RNA splicing patterns, either directly or indirectly, may have the potential to delay or reverse cellular senescence with consequent positive impact on human health span.

## Results

### Synthesis of novel resveralogues

**Fig. 1:**
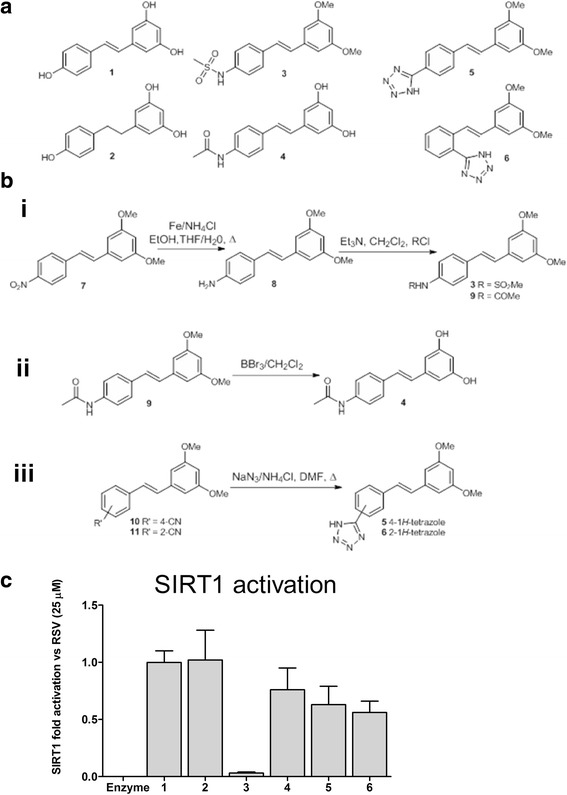
Synthesis and characterisation of novel resveralogues. **a** Structures of resveralogues 1–6. Compounds are: **1** resveratrol, **2** resveratrol’s primary metabolite, dihydroresveratrol, **3** (*E*)-N-(4-(3,5-dimethoxystyryl) phenyl)methanesulfonamide, **4** (*E*)-N-(4-(3,5-dihydroxystyryl)phenyl)acetamide, **5** (*E*)-5-(4-(3,5-dimethoxystyryl)phenyl)-1*H*–tetrazole and **6** (*E*)-5-(2-(3,5-dimethoxystyryl)phenyl)-1*H*–tetrazole. **b** Scheme of synthesis of compounds **3–6** (see Methods for details). **c** Fluorescence determination of SIRT1 activity in vitro in the presence of 25 μM each compound, normalised against resveratrol (1) and vehicle only control (0). Data are presented as fold change (mean ± SD) in activity normalised to enzyme-only (0) and resveratrol (1), such that 0 represents no activation, and 1.0 indicates activation equivalent to that observed with resveratrol 1. The experiment was carried out in 3 replicates. The numbers on the X axis (**1–6**) refer to the identity of each resveralogue as indicated above. Uncertainty was calculated by subjecting the standard deviation of the control, Resveratrol and compound data to combination using standard methods for propagation of uncertainty [49]

Resveratrol (RSV) has been reported to extend lifespan in various model organisms through activation of the NAD-dependent protein deacetylase, SIRT1 [24], while replenishment of NAD^+^ improves lifespan and health span in ATM^−^ worms and mice [25]. We therefore set out to rationally design a panel of novel resveratrol-like compounds (Fig. 1a) with the goal of identifying compounds that could restore splicing factor expression to levels comparable with those seen in young cells, but with differing effects on SIRT1 activation and the senescence-associated secretory phenotype (SASP) to allow assessment of molecular mechanism. Synthesis of the backbone was achieved as previously reported [26], with additional functionality and diversity achieved via functional group interconversion (Fig. 1b). Compounds were chosen for further analysis based on (i) structural novelty and low cytotoxicity (ii) differential SIRT1 activation activity (iii) differential effects on the suppression of SASP components and (iv) previously observed increases in the Ki67 positive fraction of MRC5 cultures at 5 μM. We also included the parent compound (resveratrol) and a major metabolite (dihydroresveratrol).

### SIRT1 activation is significantly altered following side chain modification of resveratrol

**Fig. 2:**
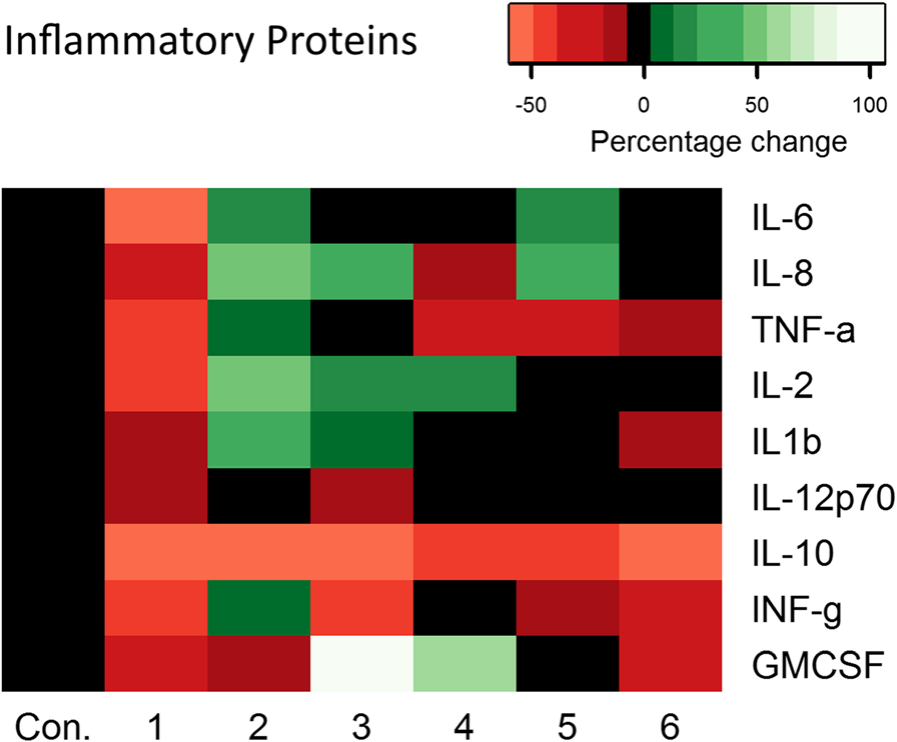
Differential effects of resveralogues on the senescence-associated secretory phenotype (SASP). Protein levels of various pro-inflammatory SASP factors was determined using Mesoscale ELISA platform in culture medium of senescent HNDF cultures treated with 5 μM resveralogues 1–6. The heat map indicates fold changes. Con = control (vehicle only). Green indicates up-regulation while red denotes down-regulation. The colour scale refers to percentage change in expression. Experiments were carried out in duplicate a total of 10 times

Since RSV has been suggested to exert its pro-longevity effects predominantly through activation of SIRT1, we first tested the ability of our novel compounds to activate SIRT1 in an ex vivo enzyme assay (Fig. 1c), with data normalised against activity detected on treatment with resveratrol (RSV, **1**). While dihydroresveratrol (Fig. 1a, 2**)** displayed SIRT1-activation activity equivalent to that of resveratrol, the four novel analogues (**3–6**) displayed a range of activities from zero (compound **3**) to around 75% of control levels (compound **4**) (Fig. 1c). These marked differences in SIRT1 activation by the novel resveralogues (compared with RSV and DHRSV) therefore allow us to probe SIRT1-dependence of any biological effects.

### Impact of resveralogues on the senescence-associated secretory phenotype

We then set out to determine if treatment with resveratrol or the novel resveralogues had an impact on the senescence-associated secretory phenotype (SASP) in senescent cultures of human fibroblasts (NHDF). The levels of multiple cytokines including key SASP components (IL6, IL8, TNFα, IL2, IL1β, IL-12p70, IL10, INFγ and GMCSF) were determined in senescent NHDF by ELISA (Fig. 2). Although each of the compounds altered cytokine profiles to some extent (Fig. 2, see also Additional file 1: Table S1), there was no consistent pattern with which this occurred. Resveratrol **1** was the only compound to reduce the levels of multiple cytokines including the key SASP mediators IL-6 and IL-8 as well as IL2, TNFα and IFNγ, consistent with previous reports [27]. By contrast, dihydroresveratrol **(2)** treatment significantly elevated levels of IL-8 and several other inflammatory mediators, whilst **3–6** had variable impact on the expression of the SASP proteins assayed. The only cytokine showing a consistent reduction in level in response to all 6 compounds was IL-10 (Fig. 2, Additional file 1: Table S1).

### Splicing factor expression and splicing patterns of senescence-associated genes are restored in senescent cultures of fibroblasts following treatment with resveralogues

**Fig. 3:**
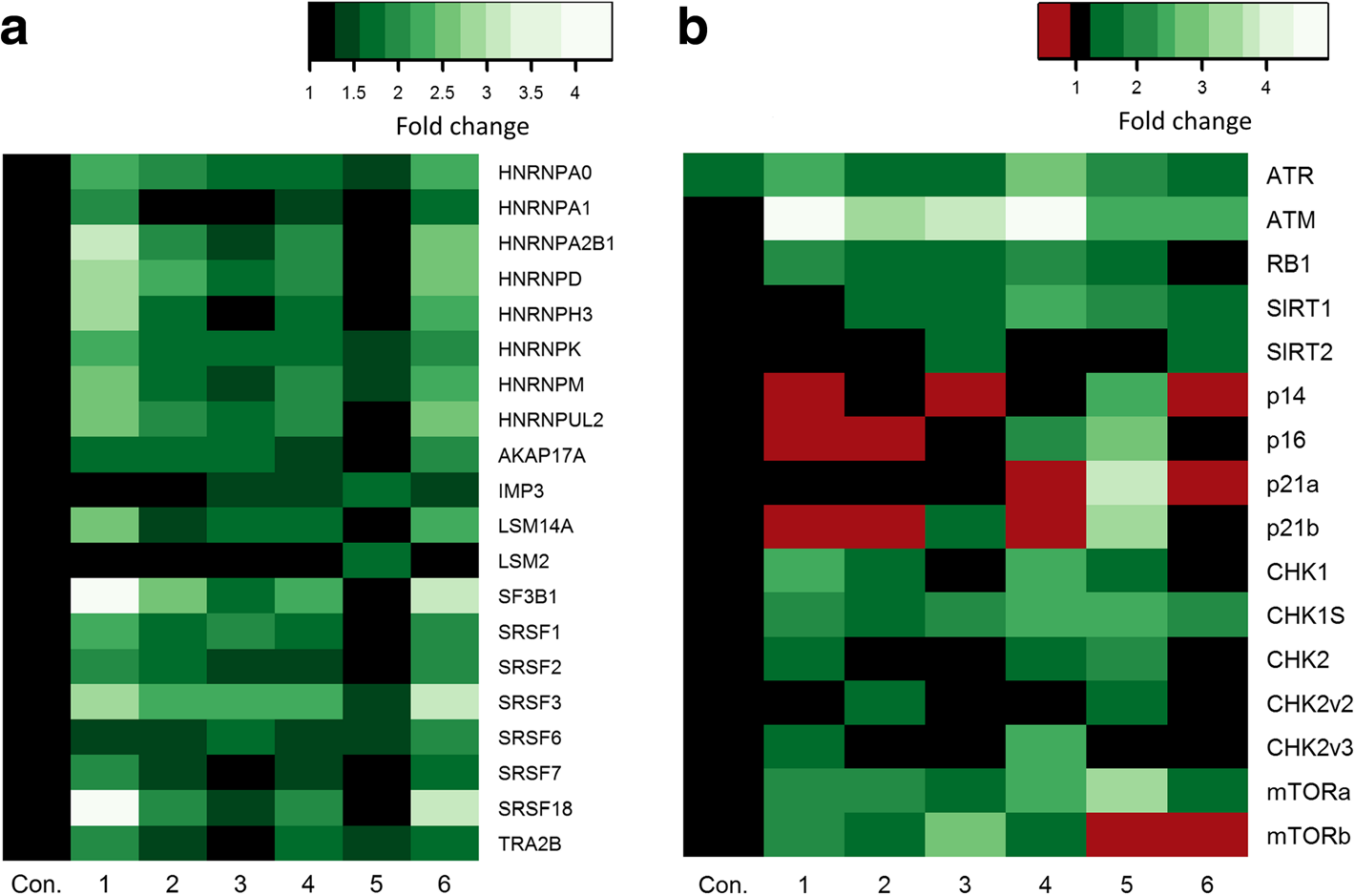
Splicing factor regulators are elevated following treatment with resveratrol analogues. Changes to mRNA levels in HNDF cells in response to treatment with 5 μM resveratrol (**1**) or resveralogues **2–6** determined by quantitative reverse transcription PCR. **a** Expression of splicing factor regulatory genes (**b**) Isoform-specific transcripts of genes associated with senescence and/or DNA damage responses. Con = control (vehicle only). Green indicates up-regulated genes, red denotes down-regulated genes. The colour scale refers to fold-change in expression. Data are derived from duplicate testing of 3 biological replicates

To establish whether RSV and the novel resveralogues could influence splicing regulators, we first measured splicing factor expression by qRT-PCR in senescent cultures of human fibroblasts (NHDF) following 24 h treatment with 5 μM of compounds **1–6**. Consistent with previous studies in HEK293 cells [22], we find that resveratrol (**1**) treatment increased levels of both splicing activators (*SRSF* transcripts) and inhibitors (*HNRNP* transcripts) (Fig. 3a). Importantly, novel resveratrol analogues also partially restored levels of both splicing activator and inhibitor transcripts (Fig. 3a, Additional file 2: Table S2). The level of restoration of splicing regulator expression in treated cells was similar to levels previously reported in early passage fibroblasts [2]. This reversal of the age-related decline in splicing factor expression was present for compounds with no discernible SIRT activity (compound **3**) as well as those that elevated IL6 and IL8 levels (compounds **2** and **5**), indicating that the action of splicing factors is independent of SIRT1 and the SASP.

We then asked whether this restoration of a ‘youthful’ complement of splicing factors is biologically relevant. To do this, we examined the alternative splicing profiles of key genes involved in cellular senescence in senescent NHDF cultures treated with each of the compounds (Fig. 3b). In some cases, it was not possible to distinguish an effect on splicing from effects on transcription, since multiple isoforms were affected with the same directionality. For example, both *p14ARF* and *p16INK4A* isoforms of the *CDKN2A* gene, which increases with cellular senescence [28], were down-regulated in response to treatment with most resveralogues. However, in other cases, only one isoform was affected; the expression of the pro-apoptotic p21b isoform, but not the consensus isoform p21a of the *CDKN1A* gene was altered, demonstrating an effect on splicing. Similarly, increased expression of the *CHK1S* isoform of the *CHK1* gene, which induces mitosis [29], but not the consensus *CHK1* isoform which does not, was seen (Additional file 2: Table S2). *SIRT1* mRNA expression was upregulated by treatment with the novel resveralogues but not RSV itself. A major regulator of cell proliferation and potential driver of senescence is mTOR: inhibition of mTORC1 by rapamycin increases longevity in animal models [30], while mTORC inhibition can reverse multiple phenotypes of cell senescence [31]. We found elevated expression of both the *mTORα* and β isoforms, which regulate cell metabolism and cell proliferation respectively [32], on treatment with resveralogues **1–4** (Fig. 3b, Additional file 2: Table S2), though mTORβ was suppressed on exposure of cells to resveralogues **5** and **6**. Overall, the changes in alternatively-expressed isoforms following resveralogue treatment are consistent with a shift towards a more proliferation-competent repertoire.

### Treatment of senescent cells with resveralogues is associated with reduction in biomarkers of senescence

**Fig. 4:**
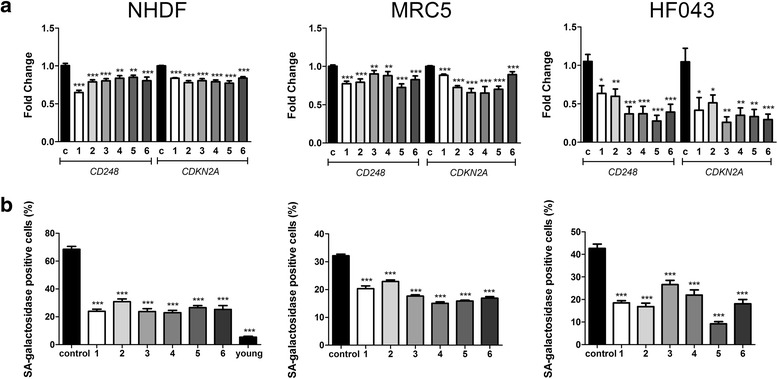
Decreased senescence biomarkers on resveralogue treatment (**a**) Levels of senescence-associated transcripts *CDKN2A* and *CD248* were assessed in senescent populations of NHDF, MRC5 and HF043 fibroblasts by quantitative reverse transcription PCR. Data are expressed relative to stable endogenous control genes GUSB, IDH3B and PPIA, and normalised to the levels of the individual transcripts in untreated controls (c), 1–6 = resveralogues **1–6**. Fold change was calculated for in triplicate for three biological replicates (**b**) Senescence associated β-galactosidase following treatment with resveralogues **1–6** was determined by manually counting the percentage of SA-β gal positive cells (NHDF, MRC5 and HF043) in each treated or control population. *n* > 300 for each sample. Statistical significance is indicated by * = *p* < 0.05, ** = *p* < 0.005, *** = *p* < 0.0005 (2 way ANOVA)

To assess whether restored splicing factor expression was associated with rescue from cellular senescence, we treated senescent cultures of normal human diploid fibroblasts from three genetically distinct cell strains (NHDF and HF043 dermal fibroblasts and MRC5 lung fibroblasts) for 24 h with compounds **3–6,** compared with RSV **(1)** and DHRSV **(2)** and measured transcript levels of senescence biomarkers *CD248* and *CDKN2A* (encoding p16^INK4/6^) by quantitative reverse transcription PCR, normalised against the *IDH3B*, *GUSB* and *PPIA* endogenous control genes, which we have found to be stable in response to senescence and ageing in our previous work [1, 2]. Stability of control genes to resveralogue treatment was verified empirically. While we observed differences between the cell lineages, there was an overall significant decrease in *CDKN2A* and *CD248* molecular markers of senescence compared with vehicle-only control cell populations (Fig. 4a), which was most marked for the foreskin fibroblast line HF043. To further assess senescence, we examined levels of senescence-associated β galactosidase (SA β-Gal). The percentage of NHDF cells staining positive for SA β-Gal decreased from ~75 to ~25%, compared with much lower levels (~7%) in younger cells at PD25 (Fig. 4b), and similar highly significant reductions in SA β-Gal reactivity were seen in senescent cultures of MRC5 and HF043 fibroblasts (Fig. 4b). These reductions in senescence markers were still evident in NHDF cells 4 weeks after initial treatment and larger reductions occurred following repeated treatments at 48 h intervals (Additional file 3: Figure S1). We conclude therefore that senescence markers are markedly diminished upon resveralogue treatment. Given that compound **3** (which does not activate SIRT1) has very similar effects on these senescence biomarkers compared with resveratrol and other resveralogues with variable SIRT-activation activity (**4, 5, 6**), we can conclude that the decrease in senescence biomarker expression on resveralogue treatment can occur independently of SIRT1 activation.

### Treatment of senescent cells with resveralogues is associated with re-entry of cell cycle

**Fig. 5:**
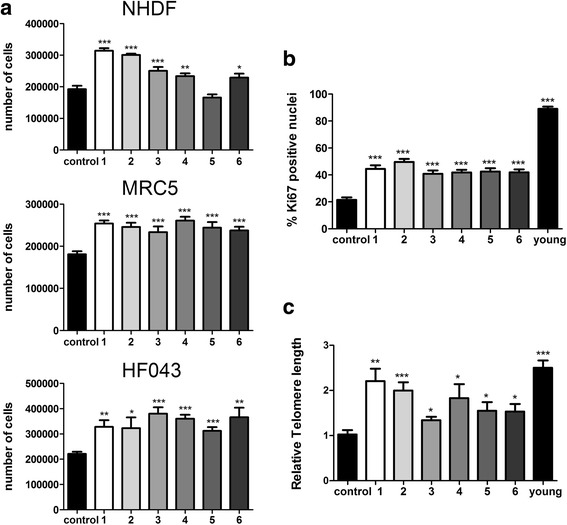
Increased proliferation of senescent cell populations following resveralogue treatment. **a** Cell numbers of NHDF, MRC5 and HF043 fibroblast populations following treatment with resveralogues **1–6**. Experiments were carried out in triplicate for three biological replicates and *** represents *p* < 0.001 (2 way ANOVA). **b** Proliferation index was assessed for control and treated NHDFs, as well as younger (PD25) cells as assessed by Ki67 immunofluorescence (> 400 nuclei counted per sample, *** *p* < 0.001 by 2 way ANOVA). **c** Telomere length was quantified by qPCR relative to the *36B4* endogenous control and normalised to telomere length in vehicle-only controls, younger passage cells (PD25) and in cells treated with compounds **1–6**. Experiments were carried out in triplicate for three biological replicates. Statistical significance is indicated by * = *p* < 0.05, ** = *p* < 0.005, *** = *p* < 0.0005 (2 way ANOVA)

While decreases in senescence biomarkers may be beneficial in alleviating some of the detrimental effects of senescent cells, it is the loss of proliferative capacity of senescent cell populations that is likely to lead to stem cell exhaustion and loss of tissue function/frailty with increasing age [33]. We therefore also assessed cell proliferation and re-entry into the proliferative cell cycle. Initially, using live cell imaging of senescent NHDF cells treated with resveratrol for up to 92 h, we found that some cells within this population showed clear evidence of mitosis within a little as 17.5 h after treatment (Additional file 4: Figure S2). We therefore assessed whether senescent populations of three different fibroblasts lines (NHDF, MRC5 and HF043) could undergo mitosis following treatment with the novel compounds. Remarkably, treatment with even very low doses (5 μM) of the resveralogues led to significant increases (up to 0.6 population doublings) in total cell numbers over only 24 h of drug exposure, while vehicle-only controls remained proliferation-arrested (Fig. 5a). Increases in cell number strongly suggest that a significant proportion of cells in the non-cycling senescent population have been induced to re-enter the mitotic cell cycle.

### Cell proliferation kinetics are altered in treated cells

To further probe this potential induction of proliferation, the proliferation kinetics of these cultures were determined by immunocytochemical and catalytic histochemical measurement of the levels of the proliferation marker, Ki67, and the senescence marker, SA β-Gal, respectively. Compounds **1–6** induced a consistent increase in the Ki67 positive fraction of cells in senescent NHDF cultures from ~20% of nuclei to ~40%, whereas levels in younger cells at PD25 were > 90% (Fig. 5b), consistent with the findings of increased cell numbers and mitotic figures following drug administration (Fig. 5a, Additional file 4: Figure S2 and data not shown). Since the increased number of cells staining for the proliferation marker Ki67 correlates inversely with the decreased numbers staining for SA-β gal (see Fig. 4b), we suggest that cells have exited senescence to enter the cell cycle.

### Treatment of senescent cells with resveralogues is associated with telomere elongation

Telomere shortening is perhaps the best known trigger of cellular senescence. Several splicing factors have been previously demonstrated to unwind telomeres and activate telomerase and could thus potentially lengthen telomeres [13, 14, 34]. We therefore measured telomere length by qPCR in NHDF cells treated with 5 μM resveratrol or resveralogues for 24 h, relative to telomere length in untreated cells. We found that cells treated with resveratrol or any of the novel resveralogues had telomeres that were 1.3–2.4 times longer than vehicle-only controls, compared with younger cells at PD25, which showed telomeres 2.6 times longer than untreated senescent cells (Fig. 5c).

### Changes in splicing factor expression and senescence markers are not effects of cell proliferation

**Fig. 6:**
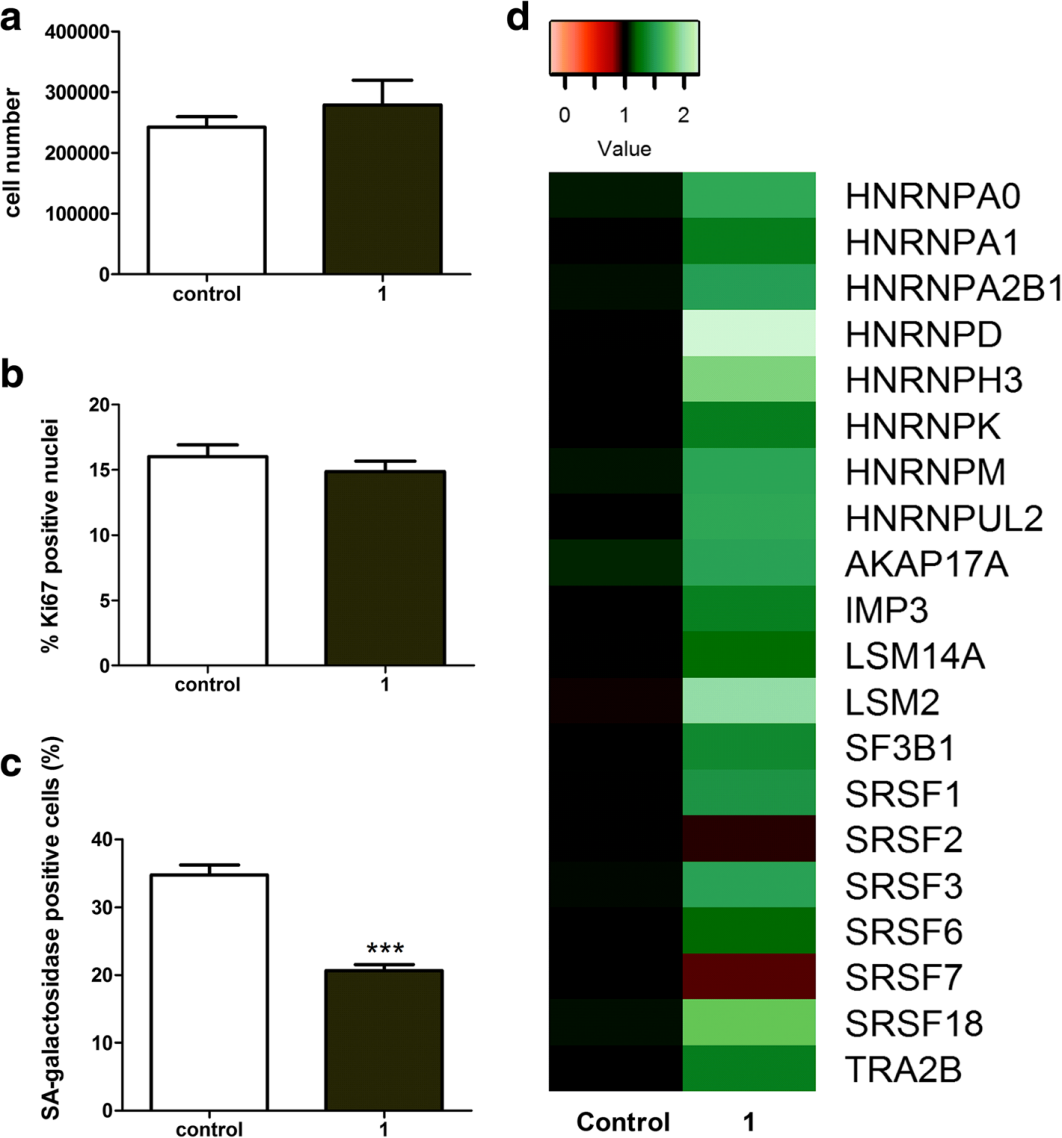
Effects of resveratrol treatment in cells grown under serum starvation conditions. **a** Cell numbers of NHDF fibroblasts following treatment with 5 μM resveratrol for 24 h under conditions of serum starvation. Experiments were carried out in triplicate for three biological replicates. (2 way ANOVA). **b** Proliferation index was assessed for NHDF fibroblasts following treatment with 5 μM resveratrol for 24 h under conditions of serum starvation as assessed by Ki67 immunofluorescence (> 400 nuclei counted per sample). **c** Senescence associated β-galactosidase following NHDF fibroblasts following treatment with 5 mM resveratrol for 24 h under conditions of serum starvation was determined by manually counting the percentage of SA-β gal positive cells in each treated or control population. *n* > 300 for each sample. Statistical significance is indicated by *** = *p* < 0.0005 (2 way ANOVA). **d** Changes to splicing factor mRNA levels in NHDF fibroblasts following treatment with 5 μM resveratrol for 24 h under conditions of serum starvation determined by qRTPCR. Control = vehicle only. Green indicates up-regulated genes, red denotes down-regulated genes. The colour scale refers to fold-change in expression. Data are derived from duplicate testing of 3 biological replicates

To determine whether the changes in splicing factor expression were a cause or consequence of renewed cell proliferation, we measured splicing factor expression and selected senescence markers under low serum conditions, which would induce proliferating cells to enter quiescence. Unsurprisingly, serum-starved cultures demonstrated no increase in cellular proliferation in response to resveralogue treatment, as determined by lack of an observable increase in cell numbers (Fig. 6a) or Ki67 index (Fig. 6b) in treated cells. However effects on both senescence markers (Fig. 6c) and splicing factor expression (Fig. 6d) were still observed, indicating that the effects on senescence and splicing factor expression were independent of proliferation. Uncoupling rescue from proliferation also allows us to quantify more precisely the percentage of cells in which senescence has been reversed from the dilution effect of increased cell number. The number of ‘reverted’ cells is ~15%, which is similar to the levels we had predicted based on the cell proliferation kinetics.

### Decrease in senescent cell fraction is not due to selective death of senescent cells

To exclude the possibility that the decrease in the percentage of senescent cells following treatment resulted from selective cell death of non-proliferating cells, cytotoxicity was assessed using an assay for extracellular lactate dehydrogenase (LDH); this intracellular enzyme is only released into the culture medium upon cell death. In all cases, cells treated with the novel compounds released lower levels of LDH than those treated with RSV (at doses up to 100 μM) in comparison with vehicle only controls (Additional file 5: Figure S3); compound **6** in particular showed very low levels of LDH release. These results demonstrate low cytotoxicity of dihydroresveratrol and all four novel resveralogues.

While necrotic cell death was not detected, it was important to rule out selective loss of senescent cells by apoptosis. Levels of apoptosis in senescent NHDF cultures treated with resveralogues **1–6** were determined by both TUNEL and by Caspase 3 and 7 assays (Additional file 5: Figure S3B and C). No increases in levels of apoptosis were observed in the resveralogue-treated cultures compared with vehicle-only control treatments, suggesting that the increased proliferation on resveralogue treatment was not a consequence of selective death of non-proliferating cells within the population.

### ERK agonists and antagonists influence cellular senescence and splicing factor expression

ERK signalling has previously been reported to be influenced by resveratrol [35, 36]. ETS-1, a transcription factor downstream of ERK activation has also been reported to regulate the expression of *TRA2B*, an important splicing regulator [37]. To investigate the potential interplay between resveratrol and ERK signalling on splicing factor expression and cellular senescence phenotypes, we treated senescent NHDF cells with low dose (1 μM or 10 μM) of trametinib, a well-characterised signalling inhibitor that inhibits the ERK signalling pathway. Treatment of senescent cells with trametinib resulted in a robust decrease in the proportion of senescent cells in the culture, which was apparent at 1 μM and 10 μM, but not at 20 μM. Such dose effects are not uncommon in signalling pathways due to interconnectivity with other signalling pathways and autoregulation (Additional file 6: Figure S4A). Conversely, treatment with the ERK agonist ceramide resulted in a comparable increase in the senescent cell fraction after 24 h. Notably, the effect of ceramide was negated by the addition of 5 μM of any of the novel resveralogues (Additional file 6: Figure S4B). Trametinib also restored splicing factor expression to profiles consistent with earlier passage in a manner similar to that observed with the resveralogues (Additional file 7: Figure S5).

## Discussion

We have generated a panel of novel molecules based on the small molecule resveratrol, to determine whether alteration to regulators of mRNA processing could influence cellular senescence phenotypes in human fibroblasts of different lineages. Treatment of senescent cultures of cells from different genetic backgrounds with these novel molecules was associated with an increase in the expression of multiple splicing factors, to levels consistent with those seen in early passage cells [2], although at present it is not clear whether these are direct or indirect effects. Treatment with all 6 resveralogues also resulted in a decline in the senescent cell fraction, along with changes to the splicing patterns of genes involved in cell senescence to a profile indicative of much ‘younger’ cells. Our evidence suggests cells have also re-entered the cell cycle, as determined by an increase in markers of cell division with concurrent increases in cell number. Finally, in accordance with the reported role of some splicing factors on telomere accessibility and telomerase activity [13–15], telomere length was lengthened in treated cells, consistent with a ‘resetting’ of the telomere clock. The absence of any elevation of either necrotic (LDH release) or apoptotic cell death (TUNEL and caspase), also excludes the possibility that the dramatic decline in the senescent fraction results from selective killing of senescent cells by resveralogues.

Disruption to splicing factor transcript expression levels is known to be a major feature of ageing in humans [1] and also in senescent human primary cell lines of multiple lineages which have undergone in ‘ageing’ by repeated culture in vitro [2]. Splicing factor expression is also associated with lifespan in humans and also in mice, where their expression appears to be early-life determinants of longevity [3]. Recent data adds weight to this hypothesis, since abolition of the core splicing factor 1 (SFA-1) alone was to reduce lifespan in *C.elegans* by interaction with the TORC1 pathway [4]. Splicing factors are also known to be drivers of cell proliferation [38–40], through effects both on splicing patterns, and through their non-canonical roles in telomere maintenance [13–15, 17, 34]. Telomere maintenance is critical in permitting cell proliferation; restoration of hTERT allows prematurely senescing human Werner syndrome fibroblasts to proliferate with kinetics of wild type cells [41]. Splicing factors hnRNPK and hnRNPD interact with the hTERT promoter while knockdown of hnRNPD notably reduces transcription of the telomerase gene [13, 14]. Additionally, hnRNPA1 is required for telomere maintenance in multiple species and has been proposed to facilitate the access of telomerase to the telomere [15].

The question of whether senescence drives splicing changes, or whether splicing alterations are causative of cell senescence in different species, tissues and points in the life course is a challenging and multifaceted one. The conventional approaches to answer this question are intractable in this system, since there are over 100 splicing factors involved in regulation of splicing, with exon usage determined by the balance of activators and inhibitors at each individual splice site [10]. The pattern and dosage of splicing factors involved will also differ from splice site to splice site and from tissue to tissue. There is also redundancy between splicing factors, both in terms of regulation of splicing, and also in their non-canonical roles - at least 6 hnRNPs and some SRSF proteins are known to have effects on telomere structure or telomerase activity. However, in this initial study the issue of causality can be distilled down to whether the changes in splicing factor expression we observe on resveralogue treatment drive rescue from senescence or are a consequence of re-entry into cell cycle. The observation that the alteration in splicing factor expression and the decrease in numbers of senescent cells occurs when proliferation is blocked provides evidence to suggest effects we note may lie upstream in the causal pathway (Fig. 5).

Presently, it is not possible to attribute specific splicing changes to alterations in the levels of specific splicing factors. Splice site choice is governed by the balance of activators and inhibitors at individual splice sites, and the binding sites are short and degenerate [10]. Similarly, in some cases, it is not possible to determine whether the effects on expression we note are transcriptional or due to splicing on the basis that expression changes of alternative isoforms share directionality. However, for other genes, the effect is confined to specific isoforms, clearly indicating an effect on splice site choice. Another caveat to our work is that we have assessed expression changes at the level of mRNA only. This is due to the inherent difficulty in culturing sufficient quantities of senescent cells to allow large scale protein analysis.

At present, the specific mechanism(s) by which resveralogues may influence splicing factor expression and senescence phenotypes in our work are not clear. Resveratrol has previously been demonstrated to have beneficial effects on senescence phenotypes through other pathways such as SIRT1 activity [24] and also through effects on the senescence-associated secretory phenotype (SASP) [23]. Our data suggest that resveralogues can influence splicing factor expression and cell division in senescent cultures independently of SIRT1 activity, since one compound, molecule **3** has no discernible SIRT1 activity (Fig. 1), despite an induction of *SIRT1* at the mRNA level. The action of resveratrol on SIRT1 is at the level of enzyme activation. In the case of compound 3, although there appears to be an effect on transcription, this compound is not able to activate the translated protein. Our data are also consistent with earlier studies in siRNA SIRT1 knockout cells which demonstrated that the effect of resveratrol on splicing factor expression occurs irrespective of SIRT1 activity [22]. Similarly, although resveralogues **1**–**6** display very similar effects on splicing factor expression, ability to supress the SASP varies widely (as shown in Fig. 2). Indeed, treatment with compound **2** significantly elevates levels of IL-8, one of the canonical cytokines that causes paracrine senescence (alongside IL-6). The only consistent change to cytokine levels that we detect is a reduction in IL-10, which is not growth suppressive.

Resveratrol has been reported to modulate the ERK pathway [35, 36]. ERK signalling has previously been suggested as a potential regulator of splicing factor expression [37]. Indeed, ETS1, a downstream target of ERK signalling has previously been reported to regulate the expression of TRA2B [37]. ERK inhibition has also been demonstrated to suppress cellular senescence [42] and to influence lifespan in animal models [43]. Our data are consistent with these observations, since alterations to ERK signalling with ERK antagonists was also associated with altered splicing factor expression and senescence phenotypes. ERK agonists were also able to ameliorate the effects of resveratrol on both phenotypes. At present, however, we cannot state definitively that this is the primary mode of induction of these effects, given the context and cell type dependence of ERK signalling, and the existence of crosstalk with other pathways. Interpretation of data are also made more complicated by the observation that even a population of senescent cells derived from a single ‘young’ culture is actually fairly heterogeneous, consisting of deeply senescent, newly senescent and pre-senescent cells. Within a senescent cell culture, there are also several routes by which those cells may have become senescent. These include replicative senescence, mitochondrial senescence, oncogene-induced senescence, paracrine senescence and autocrine senescence. At the present time, it is unclear whether all subpopulations respond to resveralogue treatment equivalently, or whether cells that have become senescent via different routes respond equivalently to resveralogues.

There is already considerable interest in the development of drugs that can attenuate senescence for eventual human use. Notable successes have come from overcoming apoptosis in senescence using Bcl-2 inhibitors [44], and by modifying mTORC signalling using rapamycin and other rapalogues or ATP mimetics specific for the mTOR kinase active site [31]. SIRT1 is also a current target for drug design and for nutraceutical interventions and is known also to be activated by resveratrol. We suggest that focusing on SIRT1 activity alone may be misleading and that other pathways activated by resveralogues may be more important in alleviating senescence and improving health outcomes in later life. The renewal of proliferation we observe upon resveralogue treatment obviously raises questions about the potential cancer risk attached to such treatment, should it eventually be employed in a clinical setting. We propose that the renewed proliferation arises from a transient increase in telomerase activity brought about by the induction of specific splicing factor proteins, and that the growth is still regulated. This is in accordance with observations that treatment with resveratrol has been suggested to have a protective effect against cancer in both humans and rodent models [45, 46].

## Conclusions

During the ageing process, both senescent and non-senescent cells lose a degree of response to cellular stressors. The upstream causes of this are as yet unclear, but may include changes in genes controlling alternative splicing; a major regulator of gene expression which ensures genomic plasticity. Here, we provide evidence that treatment with novel analogues of the stilbene compound resveratrol is associated not only with restoration of splicing factor expression but also with amelioration of multiple cellular senescence phenotypes in senescent human primary fibroblasts. At present, the precise mechanisms behind these observations are unclear, but may involve both the restoration of a more ‘youthful’ pattern of alternative splicing, and also effects of specific splicing factors on telomere maintenance. We propose therefore that splicing factors, and the upstream drivers of splicing factor expression may prove promising as druggable targets to ameliorate ageing phenotypes and hold promise as anti-degenerative compounds effective in human cells in the future.

## Methods

### Synthesis of novel resveralogues

Resveratrol (Sigma Aldrich, UK; **1**) was used to synthesise dihydroresveratrol **2** as reported previously [47]. (*E*)-N-(4-(3,5-Dimethoxystyryl)phenyl) methanesulfonamide **3** was synthesised from the previously reported nitro-substituted analogue **7** via an Fe/NH_4_Cl reduction to give amine **8** [26], followed by sulfonylation with methanesulfonyl chloride (Fig. 1b). The corresponding amide **9** was also prepared from **8**, by acylation with acetylchloride. The product **9** was subjected to demethylation (BBr_3_, CH_2_Cl_2_) to give the target compound (*E*)-N-(4-(3,5-dihydroxystyryl)phenyl)acetamide **4**. (*E*)-5-(4-(3,5-dimethoxystyryl)phenyl)-1*H*–tetrazole **5** and the isomeric 2-1*H*-tetrazole analogue **6** were prepared directly via acid-catalysed cycloaddition with azide ion from the 4- and 2-cyanostilbenes [26] (**10** and **11** respectively). (Fig. 1b) Details of the synthesis, purification and characterisation of the resveralogues are given in Additional file 8.

### Determination of SIRT1 enzyme activation

SIRT1 enzyme activity was measured by using the SIRT1 Fluorometric drug discovery kit (Cayman Chemicals, Michigan, USA) according to the manufacturer’s instructions. This assay is a standard direct fluorescent screening assay for SIRT1 ex-vivo and is essentially a variant of the well-known “fluor de lys” system. For determination of the relative capacity of each resveralogue to activate the enzyme, 25 μM solution of each compound (*n* = 3) was preincubated with the enzyme and co-factors before measurement of activity. Quantification was achieved by measuring output at λ_ex_ = 360 nm and λ_em_ = 460 nm. Each plate included background measurements and enzyme-only controls. Data are presented as fold change (mean ± sd) in activity normalised to enzyme-only and resveratrol **1**, such that 0 represents no activation, and 1.0 indicates activation equivalent to that observed with resveratrol **1**.

### Determination of cytotoxicity of resveralogue library

A commercial LDH release assay (Pierce LDH Cytotoxicity Assay Kit) was used to determine cell death. Briefly, MRC5 cells (at population doubling (PD) = 45) were seeded in 24 well plates at 1.3 × 10^5^ cells/cm^2^ and allowed to recover from trypsinisation for 24 h then exposed to each of the resveralogues (3 biological replicates × 3 concentrations; 10, 50 and 100 μM) for a further 24 h. 50 μl of media from each well was then mixed with an equal volume of LDH assay reaction mixture and incubated at room temperature in the dark for 30 min. 50 μl of LDH assay stop solution was added to each well and the absorbance of the solution was measured by spectrophotometry at 490 nm. Complete lysis and vehicle only positive and negative controls were included. Data are presented as mean (+/−standard deviation) % of the total lysis control.

### Culture of human primary fibroblasts (NHDF, MRC-5 and HF043)

Fibroblast cell strains of three genetic backgrounds and two lineages were used in this study: normal human dermal fibroblasts (NHDF; Heidelburg, Germany), human diploid foetal lung fibroblasts (MRC-5; Coriell Institute for Medical Research) and neonatal foreskin fibroblasts (HF043; Dundee Cell Products, UK). Standard culture conditions were a seeding density of 6 × 10^4^ cells/cm^2^ in media (C-23020, Promocell, Heidelburg, Germany) containing 1% penicillin and streptomycin, and a fibroblast-specific supplement mix consisting of foetal calf serum (3% *v*/v), recombinant fibroblast growth factor (1 ng/ml) and recombinant human insulin (5 μg/ml) (Promocell, Heidelburg, Germany). For the assays requiring senescent cultures, cells were counted and equal numbers of cells seeded at each passage until the growth of the culture slowed to less than 0.5 PD/week as previously described [2] (this occurred at PD = 64 (NHDF), 65 (MRC-5) and 64 (HF043). Viable cell numbers were determined at each passage by trypan blue staining. For cultures grown under serum starvation conditions, cells were maintained in DMEM (Sigma Aldrich, Dorset, UK) supplemented with 0.1% of serum and 1% penicillin and streptomycin in the absence of fibroblast-specific supplement, for 24 h prior to treatment.

### Quantification of secretion of key cytokines

NHDF cells from a senescent culture were seeded at 6 × 10^4^ cells/cm^2^ in a 6 well plate in serum-free media, and after 10 days were treated with 5 μM of each of **1–6** for 24 h. Cell supernatants were then harvested and stored at −80 °C. Levels of 9 cytokines (GMCSF, IFNγ, IL1β, IL2, IL6, IL8, IL10, IL-12p70, and TNFα) in cell supernatants from treated and vehicle-only control cells were determined using the K15007B MesoScale Discovery multiplex ELISA immunoassay (MSD, Rockville, USA) in 11 replicates. Proteins were quantified relative to a standard curve using a Sector Imager SI-6000 according to the manufacturer’s instructions. Data are presented as mean (+/−SEM).

### Expression profiling of splicing factor expression in cultures of senescent cells

NHDF cells were seeded at 6 × 10^4^ cells/cm^2^ in 6 well plates, allowed to grow for 10 days then treated with 5 μM of each compound for 24 h in 3 biological replicates, with vehicle only controls (DMSO). Resveratrol **1** acute treatment was at an initial dose of 5 μM, followed by culture without further treatment for 4 weeks. For chronic treatment regimes, resveratrol (or DMSO vehicle) was added once every 48 h during 4 weeks. 20 splicing factor transcripts that associated with age and replicative senescence in our previous work [1, 2] were selected a priori for assessment here. (Assay identifiers are available on request). RNA was extracted by using 1 ml of TRI reagent ® (Life Technologies, Foster City USA) according to the manufacturer’s instructions. Total RNA (100 ng) was reverse transcribed in 20 μl reactions using the Superscript III VILO kit (Life Technologies, Foster City, USA). Transcript expression was then quantified in triplicate for each biological replicate using TaqMan Low Density Array (TLDA) on the ABI-Prism 7900HT platform. Cycling conditions were 1 cycle each of 50 °C for 2 min, 94.5 °C for 10 min and then 40 cycles of 97 °C for 30 s and 57.9 °C for 1 min. The reaction mixes included 50 μl TaqMan Fast Universal PCR Mastermix (Life Technologies, Foster City, USA), 30 μl dH2O and 20 μl cDNA template. 100 μl reaction mixture was dispensed into the TLDA card chamber and centrifuged twice for 1 min at 1000 rpm to ensure correct distribution of solution to each well. Transcript expression was assessed by the Comparative Ct approach, relative to the *IDH3B*, *GUSB* and *PPIA* endogenous control genes, selected on the basis of empirical evidence for stability with age in our earlier microarray data [1] and with cellular senescence in our earlier work [2]. Transcript expression was expressed relative to the level of splicing factor expression in vehicle treated control cells.

### Assessment of total gene expression and alternative splicing for senescence-related genes

To assess gene expression and splicing, NHDF cells were seeded at 6 × 10^4^ cells/cm^2^ in 6 well plates and after 10 days were treated with 5 μM of each compound for 24 h in 3 biological replicates. Target transcripts included the known age-related genes *CDKN2A, CDKN1A, TP53, MTOR, CHK1* and *CHK2.* Probes specific to particular isoforms or groups of isoforms were designed to unique regions of the transcripts in question. Assays were validated by standard curve analysis of 7 serial 1:2 dilutions of pooled cDNA and proved robust and sensitive with an average efficiency of −3.4 and an average r^2^ for reproducibility between replicates of 0.87. PCR reactions contained 2.5 μl TaqMan Universal Mastermix (no AMPerase) (Applied Biosystems, Foster City, USA), 0.9 μM each primer, 0.25 μM probe and 0.5 μl cDNA reverse transcribed as above in a total volume of 5 μl in a 384 well plate. PCR conditions were a single cycle of 95 °C for 10 min followed by 40 cycles of 95 °C for 15 s and 60 °C for 1 min. We also measured the total expression of the *ATR, ATM. RB1, SIRT1* and *SIRT2* genes. Probe and primer details are available on request. Each biological replicate was tested in triplicate. Isoform-specific and total expression changes were examined for statistical significance by two way ANOVA analysis using SPSS v.22 (IBM, USA).

### Catalytic histochemical determination of SA β-gal positive fraction

Senescence marker SA β-Gal was assayed in triplicate using a commercial kit (Sigma Aldrich, UK); according to manufacturer’s instructions, with a minimum of 400 cells assessed per replicate.

### qRTPCR measurement of transcripts of senescence associated genes

Molecular markers of senescence (*CDKN2A* and *CD248* transcript levels) were measured by qRTPCR relative to the *GUSB* and *PPIA* endogenous control genes, on the ABI Prism 7900HT platform. PCR conditions and analysis were as previously described [2].

### Live cell capture microscopy

For live capture microscopy, cells were seeded at a density of 5 × 10^4^ cells per 35 mm glass bottomed dish (World Precision Instruments, USA) in 2 ml of media. They were then imaged on a Leica Axiovert inverted environmental microscope with heated chamber (37 °C) and CO_2_ capabilities. An image was taken every 10 min over the course of 92 h. A 20× objective was used with a 30 mS shutter speed and 10% light intensity from a widefield white light source for each image giving optimal contrast and minimal light exposure. Images were analysed using Leica LAS X software.

### Determination of cell proliferation

Senescent cultures of each strain were seeded at 6 × 10^4^ cells/cm^2^ into 6-well plates and cultured for 10 days then treated with 5 μM of each compound for 24 h. Cell counts in three replicates of treated and vehicle-only cultures were carried out manually following trypsinisation and suspension of cells and are presented as mean (+/−SEM).

### Immunocytochemical determination of Ki67 positive fraction

Proliferation index was assessed by using Ki67 staining on NHDF cells. Cells were seeded at 1 × 10^4^ cells/coverslip and after 10 days were treated with 5 μM of each compound for 24 h in 3 biological replicates. Cells were fixed for 10 min with 4% PFA and permeabilized with 0.025% Triton and 10% serum in PBS for 1 h. Cells were then incubated with a rabbit monoclonal anti-Ki67 antibody (ab16667, Abcam, UK) at 1:200 overnight at 4 °C followed by FITC-conjugated secondary goat anti-rabbit (1:400) for 1 h, and nuclei were counterstained with DAPI. Coverslips were mounted on slides in DAKO fluorescence mounting medium (S3023; Dako). The proliferation index was determined by counting the percentage of Ki67 positive cells from at least 400 nuclei from each biological replicate at 400× magnification under a Leica D4000 fluorescence microscope.

### Assessment of apoptosis using TUNEL assay

Terminal DNA breakpoints in situ 3 - hydroxy end labeling (TUNEL) was to quantify levels of apoptosis in NHDF cells. Cells were seeded at 1 × 10^4^ cells/cm^2^ in 6 well plates and after 10 days were treated with 5 μM of each compound for 24 h in 3 biological replicates. The TUNEL assay was performed with Click-iT® TUNEL Alexa Fluor® 488 Imaging Assay kit (Thermofisher, UK) following the manufacturer’s instructions. Negative and positive (DNase1) controls were also performed. The apoptotic index was determined by counting the percentage of positive cells from at least 400 nuclei from each biological replicate at 400× magnification.

### Assessment of apoptosis by assessment of Caspase 3 and 7 activity

Caspase-3 and-7 activities were assessed as secondary measures of apoptosis. Cells were seeded (1000 cells per well) in a white-walled 96-well plate and then treated with 5 μM of each compound for 24 h in 11 biological replicates alongside vehicle-only controls. Caspase-3 and -7 activities in the supernatants were then measured by Caspase-Glo 3/7 assay (Promega, Madison, WI, USA) following the manufacturer’s instructions. Luminescence was measured by using a BMG Pherastar FSX.

### Moderation of ERK signalling pathway with inhibitors and agonists

The role of ERK signalling in reversal of senescence was investigated using agonists (ceramide) and inhibitors (trametinib) of the ERK pathway. Cells from a senescent culture were seeded at 6 × 10^4^ cells/cm^2^ in a 6 well plate in serum free media, and after 10 days were treated with 1-20 μM of the ERK inhibitor trametinib (LC laboratories, Woburn, USA for 24 h hours, or with the ERK agonist N-Acetyl-D-sphingosine (C2-ceramide; Sigma Aldrich, UK) at 20 μM for 24 or 120 h. To examine the role of ERK signalling in resveralogue-induced rescue of senescence, HNDF cells were treated with 20 μM of the ERK agonist C2-ceramide as above, but with the addition of 5 μM resveralogue for 24 h.

### Assessment of telomere length in resveratrol treated cells

DNA was extracted from 2 × 10^5^ NHDF cells treated with 5 μM resveralogue for 24 h, using the PureLink® Genomic DNA Mini Kit (Invitrogen™/Thermo Fisher, MA, USA) according to the manufacturer’s instructions. DNA quality and concentration was checked by Nanodrop spectrophotometry (NanoDrop/Thermo Fisher, MA, USA). Relative telomere length was assessed by a modified qPCR protocol [48]. PCR reactions contained 1 μl EvaGreen (Solis Biodyne, Tartu, Estonia), 2 μM each primer and 25 ng DNA in a total volume of 5 μl in a 384 well plate. PCR conditions were a single cycle of 95 °C for 15 min followed by 45 cycles of 95 °C for 10 s, 60 °C for 30 s and 72 °C for 1 min. Telomere length was calculated using the comparative Ct approach relative to the *36B4* housekeeping gene and normalised to the quantification from untreated cells. Three biological replicates were tested and each was assessed in triplicate.

### Statistical analysis

Unless otherwise indicated, differences between treated and vehicle-only control cultures were assessed for statistical significance by two way ANOVA analysis using SPSS v.22 (IBM, USA).

## Additional files

Additional file 1: Table S1. Changes in inflammatory proteins following treatment with resveratrol analogues. (DOCX 13 kb)

Additional file 2: Table S2. Splicing factor expression and changes in alternative splicing following treatment with resveratrol analogues. (DOCX 19 kb)

Additional file 3: Figure S1. Changes in biochemical and molecular markers of cellular senescence following chronic or repeated treatment with resveratrol. (TIFF 154 kb)

Additional file 4: Figure S2. Live cell capture image following resveratrol treatment. (TIFF 243 kb)

Additional file 5: Figure S3. Level of necrosis and apoptosis following treatment with resveratrol analogues. (TIFF 230 kb)

Additional file 6: Figure S4. The effect of manipulation of the ERK pathway with chemical inhibitors and agonists on cellular senescence. (TIFF 193 kb)

Additional file 7: Figure S5. The effect of ERK inhibition on splicing factor expression. (TIFF 143 kb)

Additional file 8: Synthesis and characterisation of resveralogues. (PDF 3019 kb)

## Abbreviations

DHRSV: Dihydroresveratrol
ESE: Exon splicing enhancer
ESS: Exon splicing silencer
HF043: Neonatal foreskin fibroblast
hnRNP: heterogeneous ribonucleoprotein
ISE: Intron splicing enhancer
ISS: Intron splicing silencer
LDH: Lactate dehydrogenase
MRC5: Human diploid foetal lung
NHDF: Normal human dermal fibroblast
RSV: Resveratrol
SA β-Gal: Senescence-associated β galactosidase
SASP: Senescence associated secretory phenotype
SR: Serine arginine class
TLDA: TaqMan low density array
