# Supplementary material for: Small molecule modulation of splicing factor expression is associated with rescue from cellular senescence
Source: BMC Cell Biol. 2017 Oct 17;18:31. doi: 10.1186/s12860-017-0147-7 (PMC5645932; doi:10.1186/s12860-017-0147-7)
Supplement: Supplementary file 1 — Changes in inflammatory proteins following treatment with resveratrol analogues. (DOCX 13 kb) [file 12860_2017_147_MOESM1_ESM.docx]

|  | **Control** | **1** | **2** | **3** | **4** | **5** | **6** |
| --- | --- | --- | --- | --- | --- | --- | --- |
| **IL-6** | 12250 (1631) | **5437^***^ (668)** | 15081 (1936) | 11771 (1750) | 11815 (1825) | 14432 (2127) | 10922 (1125) |
| **IL-8** | 701.9 (84.0) | **441.8^*^ (68.9)** | **1030.0^*^ (124.9)** | 955.2 (120.4) | 595.2 (83.3) | 936.8 (148.2) | 681.3 (76.6) |
| **TNF-α** | 44.3 (5.3) | **25.6^**^ (4.0)** | 47.7 (8.4) | 41.6 (6.5) | 32.2 (4.6) | 33.0 (6.7) | 36.3 (5.2) |
| **IL-2** | 74.2 (9.3) | **44.3^*^ (6.4)** | 108.4 (16.1) | 94.3 (16.5) | 86.0 (15.6) | 69.4 (12.8) | 67.9 (9.7) |
| **IL1β** | 62.8 (12.9) | 48.2 (10.0) | 84.2 (17.9) | 66.5 (13.5) | 56.4 (10.2) | 59.4 (12.4) | 48.7 (7.0) |
| **IL-12p70** | 37.2 (5.7) | 30.9 (3.7) | 35.7 (5.4) | 29.6 (4.6) | 33.1 (4.2) | 34.9 (4.2) | 34.2 (4.8) |
| **IL-10** | 68.8 (12.8) | **32.7^*^ (6.5)** | **31.1^*^ (6.3)** | **32.9^*^ (4.5)** | **33.7^*^ (5.3)** | **39.1^*^ (7.4)** | **27.8^**^ (3.7)** |
| **INF-γ** | 361.1 (54.8) | 195.6 (23.5) | 384.4 (44.8) | 213.7 (32.2) | 326.4 (36.3) | 312.3 (41.8) | 266.9 (29.1) |
| **GMCSF** | 69.8 (7.9) | **44.8^*^ (5.9)** | 60.1 (6.9) | **144.0^*^ (20.1)** | **110.2^*^ (17.8)** | 65.46 (11.7) | **48.2^*^ (6.1)** |

**Additional file 1: Table S1: Changes in inflammatory proteins following treatment with resveratrol analogues** Changes in cytokine expression in dermal fibroblasts treated with 5μM resveratrol (RSV), Dihydroxy resveratrol (DH-RSV) or 4 resveratrol analogues (v24 – v34) for 24 hours are given in the table. Data were generated by ELISA analysis from serum-free tissue culture supernatant on the Meso Scale Discovery (MSD) multiplex immunoassay (K15007B) using a Sector Imager SI-6000 according to the manufacturer’s instructions. Standard error of the mean (SEM) is given in parentheses. Statistical significance is indicated by stars with * = p<0.05, ** = p<0.005, ***=p<0.0005 and genes showing significant associations are given in bold text. Cytokine units are pg/ml.
