# Supplementary material for: Small molecule modulation of splicing factor expression is associated with rescue from cellular senescence
Source: BMC Cell Biol. 2017 Oct 17;18:31. doi: 10.1186/s12860-017-0147-7 (PMC5645932; doi:10.1186/s12860-017-0147-7)
Supplement: Supplementary file 2 — Splicing factor expression and changes in alternative splicing following treatment with resveratrol analogues. (DOCX 19 kb) [file 12860_2017_147_MOESM2_ESM.docx]

|  | **control** | **1** | **2** | **3** | **4** | **5** | **6** |
| --- | --- | --- | --- | --- | --- | --- | --- |
| ***Splicing factors*** |  |  |  |  |  |  |  |
| ***HNRNPA0*** | 1.00 (0.02) | **2.2^**^ (0.37)** | **2.0^*^ (0.29)** | **1.8^*^ (0.17)** | **1.8^*^ (0.30)** | **1.4^*^ (0.09)** | **2.4^**^ (0.37)** |
| ***HNRNPA1*** | 1.00 (0.03) | **2.0^**^(0.26)** | **1.2^*^ (0.05)** | 1.0 (0.02) | **1.4^*^ (0.13)** | 0.8 (0.07) | **1.8^**^ (0.35)** |
| ***HNRNPA2B1*** | 1.00 (0.00) | **3.2^**^ (0.62)** | **1.9^**^ (0.21)** | **1.5^***^ (0.07)** | **2.0^**^ (0.16)** | 1.0 (0.03) | **2.5^*^ (0.52)** |
| ***HNRNPD*** | 1.00 (0.01) | **2.8^***^ (0.34)** | **2.3^***^ (0.27)** | **1.7^**^ (0.13)** | **1.9^**^ (0.23)** | 1.2 (0.18) | **2.7^**^ (0.40)** |
| ***HNRNPH3*** | 1.00 (0.01) | **2.8^*^ (0.65)** | **1.6^*^ (0.19)** | 1.1 (0.10) | **1.6^**^ (0.14)** | 0.7 (0.04) | **2.2^*^ (0.61)** |
| ***HNRNPK*** | 1.00 (0.02) | **2.3^**^ (0.30)** | **1.8^***^ (0.09)** | **1.7^***^ (0.03)** | **1.8^***^ (0.19)** | **1.3^*^ (0.13)** | **2.1^**^ (0.29)** |
| ***HNRNPM*** | 1.00 (0.05) | **2.5^*^ (0.44)** | **1.7^*^ (0.32)** | **1.4^**^ (0.15)** | **1.9^***^(0.28)** | **1.4^*^ (0.15)** | **2.2^*^ (0.52)** |
| ***HNRNPUL2*** | 1.00 (0.02) | **2.7^***^ (0.31)** | **2.1^***^ (0.26)** | **1.8^***^ (0.11)** | **2.0^***^ (0.22)** | **1.2^*^ (0.06)** | **2.5^**^ (0.40)** |
| ***AKAP17A*** | 1.01 (0.04) | **1.6^***^ (0.10)** | **1.6 ^*^(0.23)** | **1.7^**^ (0.16)** | **1.4^*^ (0.20)** | 1.2 (0.14) | **2.1^**^ (0.44)** |
| ***IMP3*** | 1.00 (0.06) | 1.0 (0.26) | 1.1 (0.21) | **1.4^**^ (0.15)** | **1.4^*^ (0.17)** | **1.8^**^ (0.41)** | 1.3 (0.24) |
| ***LSM14A*** | 1.00 (0.01) | **2.6^**^ (0.48)** | **1.5^*^ (0.28)** | **1.6^**^ (0.04)** | **1.8^**^ (0.21)** | 1.1 (0.09) | **2.3^**^ (0.43)** |
| ***LSM2*** | 1.00 (0.12) | 0.9 (0.35) | 1.1 (0.38) | 1.2 (0.26) | 1.0 (0.27) | **1.8^**^(0.41)** | **1.2^*^ (0.59)** |
| ***SF3B1*** | 1.00 (0.03) | **4.1^***^ (0.86)** | **2.5^*^ (0.27)** | **1.8^***^ (0.14)** | **2.4^***^ (0.38)** | 1.0 (0.08) | **3.0^***^ (0.59)** |
| ***SRSF1*** | 1.00 (0.02) | **2.3^*^ (0.51)** | **1.7^**^ (0.28)** | **1.9^***^ (0.01)** | **1.8^**^ (0.33)** | 1.2 (0.17) | **2.1^**^ (0.45)** |
| ***SRSF2*** | 1.00 (0.03) | **2.1^*^ (0.41)** | **1.7^*^ (0.43)** | **1.4^*^ (0.23)** | **1.3^*^ (0.26)** | **0.6^*^ (0.14)** | **1.9^**^ (0.35)** |
| ***SRSF3*** | 1.01 (0.05) | **2.9^**^ (0.57)** | **2.2^***^ (0.21)** | **2.2^**^ (0.22)** | **2.4^**^ (0.36)** | **1.4^*^ (0.29)** | **3.0^***^ (0.60)** |
| ***SRSF6*** | 1.00 (0.01) | **1.5^*^ (0.16)** | **1.5^**^ (0.12)** | **1.6^*^ (0.14)** | **1.5^*^ (0.22)** | **1.5^**^ (0.04)** | **2.1^**^ (0.31)** |
| ***SRSF7*** | 1.00 (0.01) | **1.9^*^(0.26)** | **1.4^***^ (0.03)** | 1.2 (0.09) | 1.3 (0.11) | 1.0 (0.08) | **1.7^*^ (0.25)** |
| ***SRSF18*** | 1.00 (0.03) | **4.4^**^ (0.98)** | **2.0^*^ (0.34)** | **1.5^***^ (0.26)** | **1.9^**^ (0.11)** | 1.1 (0.07) | **3.1^**^ (0.96)** |
| ***TRA2B*** | 1.00 (0.00) | **2.1^**^ (0.30)** | **1.5^***^ (0.08)** | 1.2 (0.11) | **1.6^*^(0.18)** | 1.3 (0.17) | **1.8^*^ (0.31)** |
| ***Total expression*** |  |  |  |  |  |  |  |
| ***ATR*** | 1.25 (0.05) | **2.46^***^ (0.11)** | **1.48^***^ (0.06)** | **1.46^***^(0.09)** | **2.69^**^ (0.51)** | 1.95 (0.56) | 1.25 (0.13) |
| ***ATM*** | 1.03 (0.08) | **4.94^***^ (0.57)** | **3.46^***^ (0.58)** | **3.58 ^***^(0.39)** | **4.88^**^ (1.03)** | **2.23^*^ (0.55)** | **2.35^***^(0.23)** |
| ***RB1*** | 1.01 (0.06) | **1.90^***^ (0.11)** | **1.37^**^ (0.10)** | **1.29^*^ (0.09)** | **1.98^*^ (0.42)** | 1.33 (0.33) | 1.05 (0.09) |
| ***SIRT1*** | 1.01 (0.05) | 1.22 (0.37) | **1.44^***^(0.08)** | **1.59^**^ (0.15)** | **2.40^*^ (0.55)** | 1.71 (0.48) | **1.38^*^ (0.15)** |
| ***SIRT2*** | 1.00 (0.02) | **1.19^*^ (0.07)** | 1.15 (0.09) | 1.24 (0.11) | 1.07 (0.07) | 1.18 (0.10) | 1.46 (0.25) |
| ***Isoform-specific expression*** |  |  |  |  |  |  |  |
| ***p14*** | 1.00 (0.01) | **0.73^***^ (0.06)** | **0.79^***^ (0.04)** | **0.74^***^ (0.03)** | **0.93^**^ (0.01)** | **2.31^*^ (0.47)** | **0.64^***^ (0.06)** |
| ***p16*** | 1.15 (0.09) | **0.62^**^ (0.10)** | **0.74^*^ (0.15)** | 1.06 (0.11) | **1.98^*^ (0.30)** | **2.79^**^ (0.42)** | 0.89 (0.29) |
| ***p21a*** | 1.00 (0.04) | 0.90 (0.05) | 0.84 (0.07) | 0.77 (0.11) | **0.67^***^ (0.04)** | **3.98^**^ (1.03)** | **0.49^***^ (0.05)** |
| ***p21b*** | 1.01 (0.05) | **0.71^**^ (0.06)** | **0.74^**^ (0.05)** | 1.24 (0.16) | **0.70^**^ (0.08)** | **3.18^**^ (0.75)** | 1.02 (0.20) |
| ***CHK1*** | 1.00 (0.01) | **2.20^***^ (0.27)** | 1.24 (0.13) | 1.03 (0.09) | **2.43^**^ (0.47)** | 1.33 (0.26) | 1.04 (0.05) |
| ***CHK1S*** | 1.09 (0.15) | **2.15^*^ (0.33)** | 1.67 (0.36) | **2.03^**^ (0.26)** | **2.52^**^ (0.37)** | **2.56^*^ (0.58)** | **1.74^*^ (0.17)** |
| ***CHK2*** | 1.00 (0.02) | 1.37 (0.21) | 0.93 (0.11) | 0.95 (0.04) | **1.63^*^ (0.22)** | **1.86^*^ (0.31)** | 0.99 (0.09) |
| ***CHK2v2*** | 1.04 (0.11) | 1.089 (0.29) | 1.36 (0.21) | 0.87 (0.11) | 1.23 (0.11) | **1.68^*^ (0.26)** | 0.91 (0.10) |
| ***CHK2v3*** | 1.02 (0.06) | 1.40 (0.18) | 0.91 (0.14) | 1.22 (0.16) | **2.20^*^ (0.40)** | 0.93 (0.17) | 1.21 (0.21) |
| ***mTORa*** | 1.00 (0.01) | **2.14^***^ (0.14)** | **1.87^**^ (0.24)** | **1.66^*^ (0.23)** | **2.58^***^ (0.28)** | **3.12^***^ (0.37)** | **1.32^**^ (0.08)** |
| ***mTORb*** | 1.00 (0.02) | **2.11^***^ (0.17)** | **1.39^***^ (0.04)** | **2.80^***^ (0.38)** | **1.67^***^ (0.01)** | **0.76^**^ (0.09)** | **0.71^*^ (0.14)** |

**Additional file 2: Table S2: Splicing factor expression and changes in alternative splicing following treatment with resveratrol analogues.** Changes in splicing factor expression and alternative splicing patterns in HNDF fibroblasts treated with 5μM resveratrol (**1**), Dihydroxy resveratrol (**2**), (*E*)-N-(4-(3,5-Dimethoxystyryl)phenyl) methanesulfonamide (**3**), (*E*)-N-(4-(3,5-dihydroxystyryl)phenyl) acetamide (**4**), (*E*)-5-(4-(3,5-dimethoxystyryl)phenyl)-1*H*-tetrazole (**5**) or the isomeric 2-1*H*-tetrazole analogue (**6**) for 24 hours are given above. Standard error of the mean (SEM) is given in parentheses. Statistical significance is indicated by stars with * = p<0.05, ** = p<0.005, ***=p<0.0005 and genes showing significant associations are given in bold text.
