# Supplementary figures and images for: Small molecule modulation of splicing factor expression is associated with rescue from cellular senescence
Source: BMC Cell Biol. 2017 Oct 17;18:31. doi: 10.1186/s12860-017-0147-7 (PMC5645932; doi:10.1186/s12860-017-0147-7)

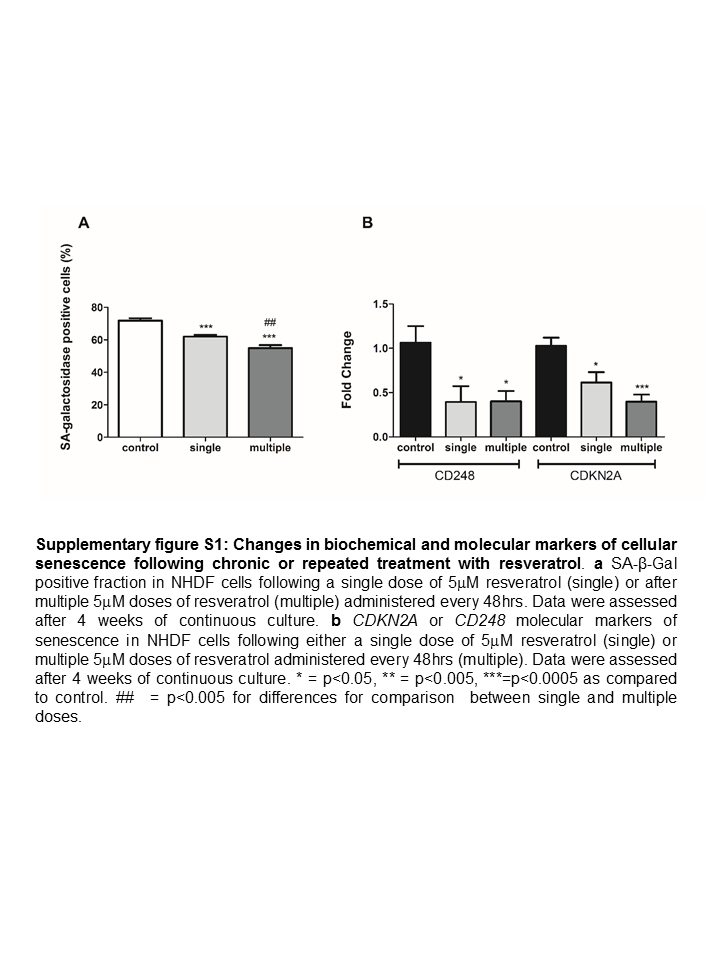

Supplement: Supplementary file 3 — Changes in biochemical and molecular markers of cellular senescence following chronic or repeated treatment with resveratrol. (TIFF 154 kb) [file 12860_2017_147_MOESM3_ESM.tif]

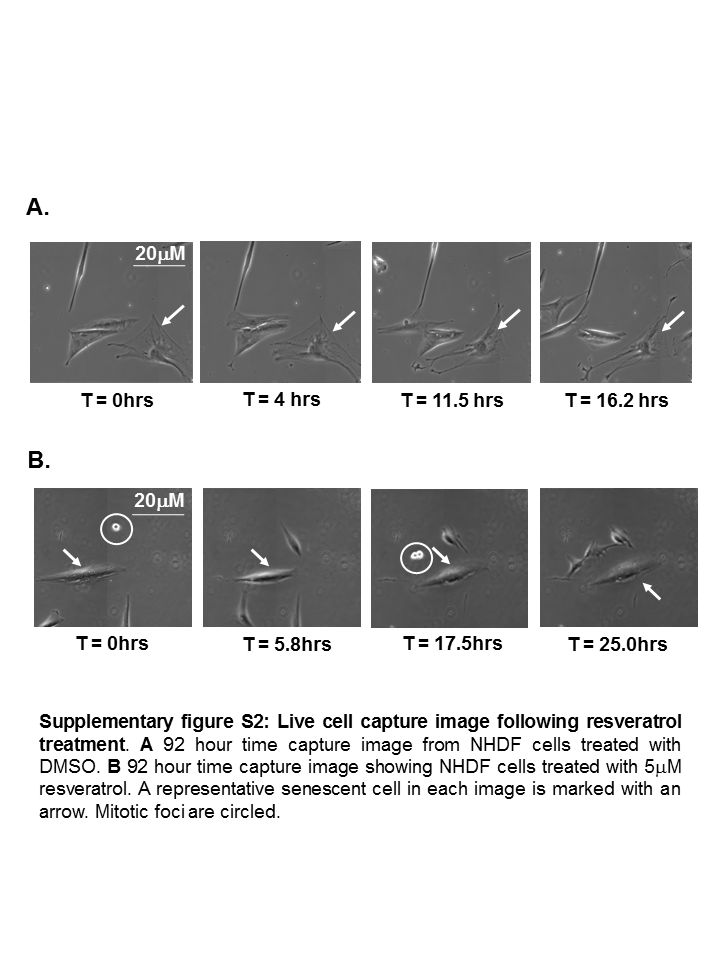

Supplement: Supplementary file 4 — Live cell capture image following resveratrol treatment. (TIFF 243 kb) [file 12860_2017_147_MOESM4_ESM.tif]

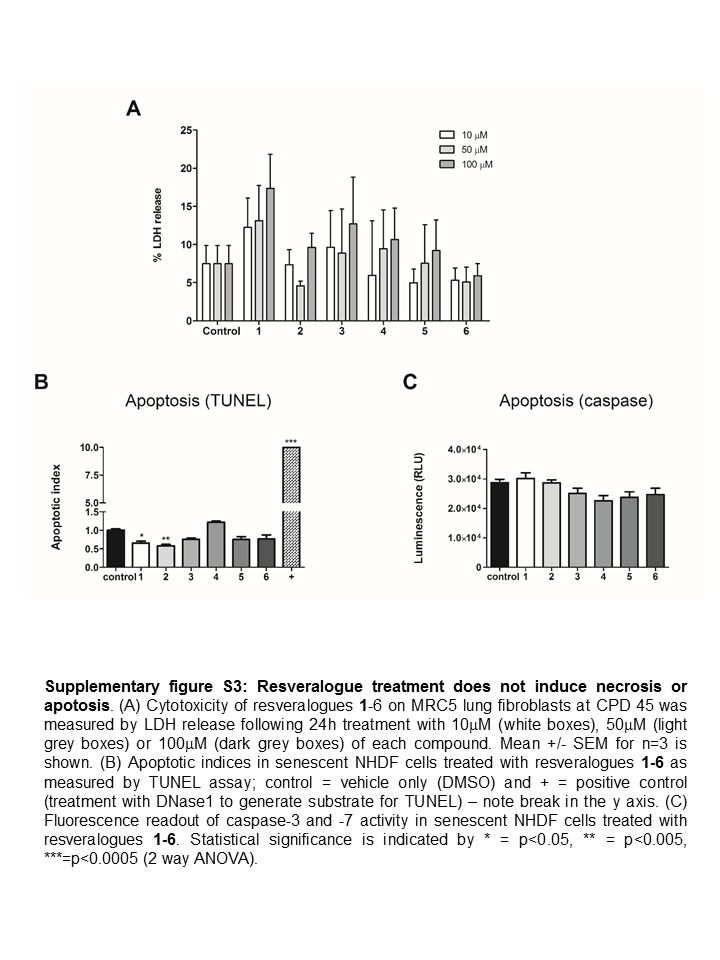

Supplement: Supplementary file 5 — Level of necrosis and apoptosis following treatment with resveratrol analogues. (TIFF 230 kb) [file 12860_2017_147_MOESM5_ESM.tif]

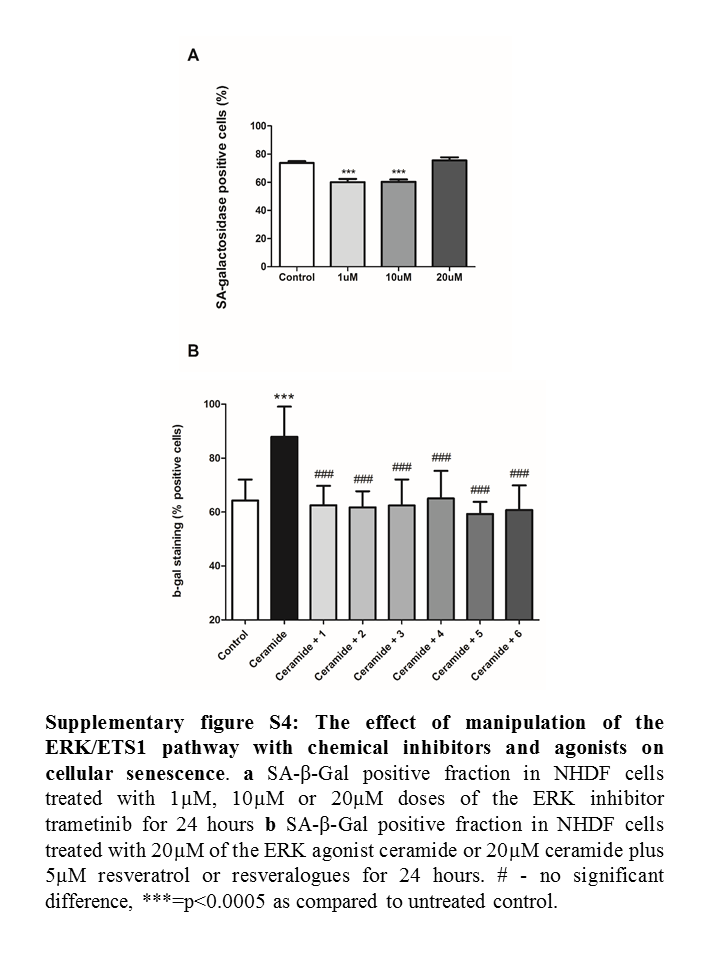

Supplement: Supplementary file 6 — The effect of manipulation of the ERK pathway with chemical inhibitors and agonists on cellular senescence. (TIFF 193 kb) [file 12860_2017_147_MOESM6_ESM.tif]

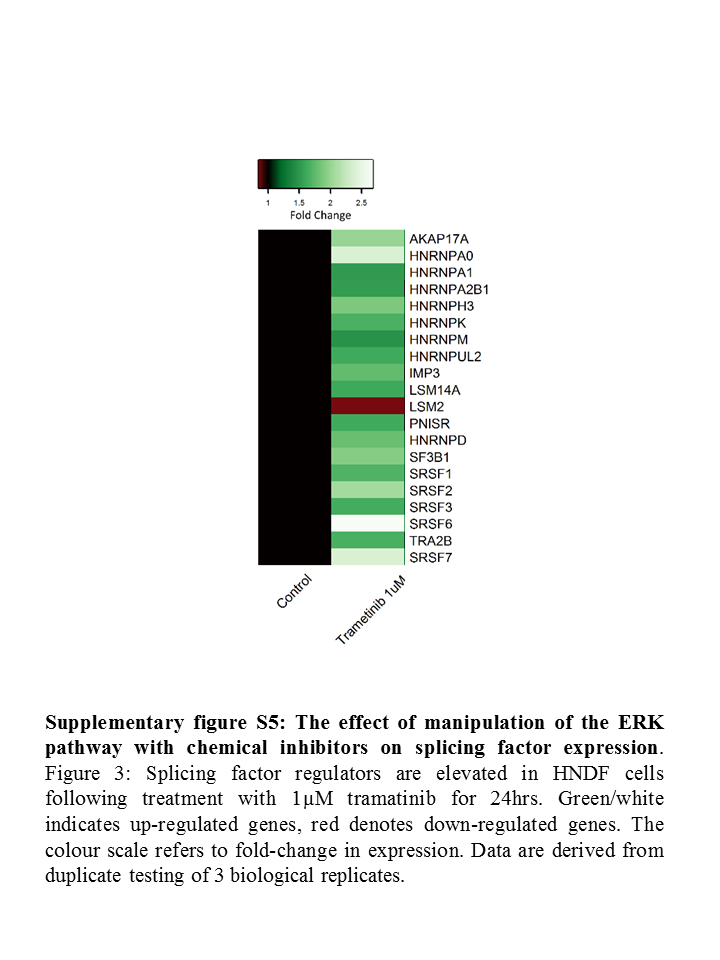

Supplement: Supplementary file 7 — The effect of ERK inhibition on splicing factor expression. (TIFF 143 kb) [file 12860_2017_147_MOESM7_ESM.tif]
