# Supplementary material for: Small molecule modulation of splicing factor expression is associated with rescue from cellular senescence
Source: BMC Cell Biol. 2017 Oct 17;18:31. doi: 10.1186/s12860-017-0147-7 (PMC5645932; doi:10.1186/s12860-017-0147-7)

SUPPLEMENTARY INFORMATION

Small molecule moderation of splicing factor expression is associated with rescue from cellular senescence

Eva Latorre[1#], Vishal C. Birar[2#], Angela N. Sheerin[2], J. Charles G Jeynes[3], Amy Hooper[1], Helen R. Dawe[4], David Melzer[1], Lynne S. Cox[5], Richard G. A. Faragher[2*], Elizabeth L. Ostler[2*] & Lorna W. Harries[1*]

* Both authors contributed equally to this work

# Co-senior authors

[1]*Institute of Biomedical and Clinical Sciences, University of Exeter Medical School, University of Exeter, Devon, UK, EX2 5DW*
[2]*School of Pharmacy and Biomolecular Sciences, University of Brighton, Brighton, UK, BN2 4GJ*
[3]*Centre for Biomedical Modelling and Analysis, University of Exeter, Devon, UK*, Ex2 5DW
[4]*College of Life and Environmental Sciences, University of Exeter, Devon, UK, EX4 4QD*
[5]*Department of Biochemistry, University of Oxford, Oxford, UK, OX1 3QU*

Contents

I. General experimental protocols 3

II. Synthetic methods and characterisation data for all new compounds 4

Synthesis of (*E*)-N-(4-(3,5-dimethoxystyryl)phenyl)methanesulfonamide (3) 4

Synthesis of (*E*)-N-(4-(3,5-dihydroxystyryl)phenyl)acetamide (4) 4

Synthesis of (*E*)-5-(4-(3,5-dimethoxystyryl)phenyl)-1*H*-tetrazole (5) 5

Synthesis (E)-5-(2-(3,5-dimethoxystyryl)phenyl)-1*H*-tetrazole (6) 6

Synthesis of (*E*)-4-(3,5-dimethoxystyryl)aniline (8) 6

Synthesis of (*E*)-N-(4-(3,5-dimethoxystyryl)phenyl)acetamide (9) 7

III. References for supplementary information 8

IV. Copies of spectra 9

NMR and Mass Spectra of 3 9

NMR and Mass Spectra of 4 12

NMR and Mass Spectra of 5 15

NMR and Mass Spectra of 6 18

NMR and Mass Spectra of 8 21

NMR and Mass Spectra of 9 23

I. General experimental protocols

All reagents and solvents were obtained commercially and used directly without further purification, unless otherwise stated. Reaction requiring anhydrous conditions were carried out in oven dried glassware under and atmosphere of nitrogen. Reactions were monitored by using thin layer chromatography performed on TLC Silica Gel 60 F254 (Merck), and visualised by irradiation with ultraviolet light (254 nm). Flash chromatography was conducted by using hand-packed silica gel (100–200 mesh) and eluent as described in the relevant method.

Proton (^{1}H) and Carbon (^{13}C) NMR spectra were recorded on a 400MHz instrument (Brüker FT-NMR). The δ values represent chemical shifts reported in parts per million (ppm), relative to internal standard tetramethylsilane (TMS), and coupling constant (*J*) values are in Hz. ^{13}C NMR spectra were definitively assigned with reference to HSQC correlation spectra. In each case the assignments are numbered as per the general structure below.

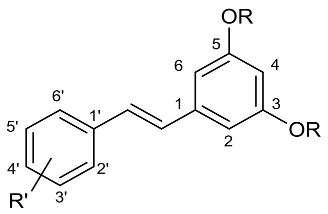

ESI-HRMS were recorded on a Brüker MicroTOF instrument. Infra-red spectra were recorded as a thin film on a Perkin-Elmer Spectrum 65. Melting points were measured in open capillaries on an Electrothermal melting point apparatus. Purity was confirmed by isocratic (methanol:ammonium acetate buffer) high performance liquid chromatography (HPLC) utilising a Kinetix 5 µm 10 cm C18 column on a Waters Alliance 2695 instrument with a Waters 2487 dual wavelength detector set to 306nm, and an injection volume of 20µl. Purity of all final compounds was 95% or higher.

II. Synthetic methods and characterisation data for all new compounds

Synthesis of (*E*)-N-(4-(3,5-dimethoxystyryl)phenyl)methanesulfonamide (3)

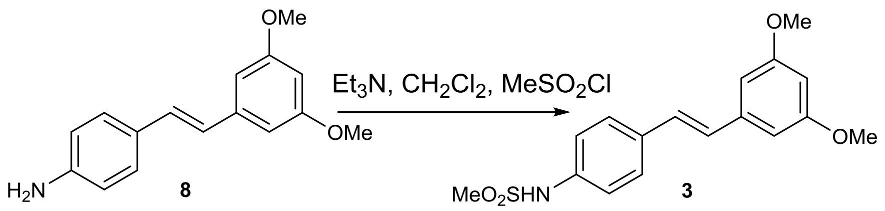

To a solution of aniline **8** (0.300 g, 3.91mmol, 1 equivalent) in dichloromethane (10 ml), triethylamine (3.1 g, 5.87 mmol, 1.5 equivalents) was added and then the mixture cooled to 0°C with stirring. After 30 min, methanesulphonyl chloride (0.399 g, 5.09 mmol, 1.3 equivalents) was slowly added and the reaction was allowed to warm slowly to RT, and was then stirred overnight. The resulting mixture was poured into water (25 ml) and extracted with EtOAc (2 x25 ml) and the combined organic layer washed with 1N hydrochloric acid (1 x 5 ml) and water (10 ml). The organic layer was evaporated under vacuum to give crude product, and this was purified by column chromatography (EtOAc:petroleum ether 2:8) to obtain the sulphonamide **3** as a yellow solid (351 mg, 90 %).

mp = 147-150 °C, Rf (10:90 EtOAc: petroleum ether) = 0.32 IR ν (cm^{-1}) = 3031, 2943, 2831, 1588, 1360, 1161, 1063, 966, 898, 757, 685. ^{1}H NMR (400 MHz, $CDCl_3$) δ (ppm) = 2.21 (3H, s, -SMe), 3.84 (6H, s, -OMe), 6.42 (1H, t, *J* = 2.1 Hz, H4), 6.67 (2H, d, *J* = 2.1 Hz, H2, H6), 7.07 (2H, s, -C=C-H), 7.34 (2H, dd, *J* = 8.4, 2 Hz, H2', H6'), 7.59 (2H, dd, *J* = 8.4, 2 Hz, H3' and H5'). ^{13}C NMR (100 MHz, $CDCl_3$) δ (ppm) = 42.7 (-SOOMe), 55.4 (OMe), 100.5 (C4), 104.9 (C2, C6), 127.4 (C=C), 127.6 (C3', C5'), 130.9 (C2', C6'), 131.2 (C=C), 132.2 (C1'), 138.6 (C1), 139.6 (C4'), 160.0 (C3, C5). HRMS m/z calculated for $C_{17}H_{18}NO_4SNa^+$ requires 355.0849; $[(M+Na-H)]^+$ found 355.06069.

Synthesis of (*E*)-N-(4-(3,5-dihydroxystyryl)phenyl)acetamide (4)

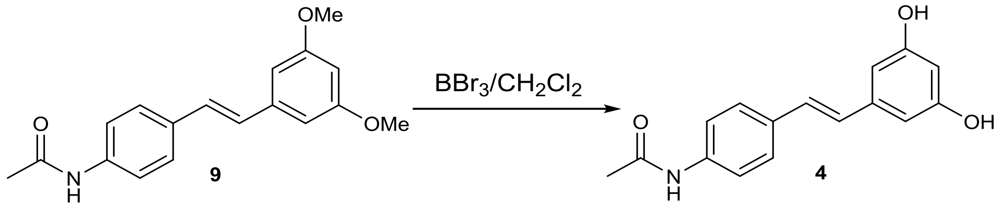

A solution of acetamide **9** (200 mg, 0.067 mmol) and 1 M BBr_3 in dichloromethane (1.0 mmol , 1 ml) in dry 1,2-dichloroethane (5 ml) was stirred at room temperature under nitrogen for 1 day. The reaction was monitored by TLC and, when complete, the mixture concentrated

under vacuum. The crude product was dissolved in EtOAc (10 ml) and washed with saturated sodium bicarbonate solution (10 ml). The organic layer was further washed with water (10 ml) and then with saturated brine (10 ml). The organic layer was dried (Na_2SO_4), filtered and evaporated and the resulting crude was purified with column chromatography (10:90 EtOAc:chloroform) to give the target dihydroxyacetamide **4** as a light brown solid (82 mg, 45 %).

mp = 112 °C, Rf (10:90 EtOAc:chloroform) = 0.4, IR ν (cm-1) = 3327, 3050, 2890, 2671, 1593, 1510, 1170, 966, 840, 631. ^{1}H NMR (400 MHz, $CDCl_3$) δ (ppm) = 2.05 (3H, s, COMe), 6.14 (1H, t, *J* = 2 Hz, H4), 6.41 (2H, d, *J* = 2 Hz, H2, H6), 6.95 (2H, s, H-C=C-H), 7.50 (2H, d, *J* = 8.8 Hz, H2’, H6’), 7.57 (2H, d, *J* = 8.8 Hz, H3’, H5’), 9.22 (2H, s, -OH), 9.98 (1H, s, NH). ^{13}C NMR (100 MHz, $CDCl_3$) δ (ppm) = 24.0 (N-Me), 102.1 (C4), 104.5 (C2, C6), 119.0 (C3’, C5’), 126.9 (C2’, C6’), 127.4 (2 x C=C), 131.7 (C1), 138.7 (C1’), 138.9 (C4’), 158.5 (C3, C5), 168.2 (CO), HRMS m/z calculated for $C_{16}H_{15}NNaO_3^+$ requires 292.0944; found [(M+Na)]+ 292.07937 and [(2M+Na)]+ 561.18057.

Synthesis of (*E*)-5-(4-(3,5-dimethoxystyryl)phenyl)-1*H*-tetrazole (5)

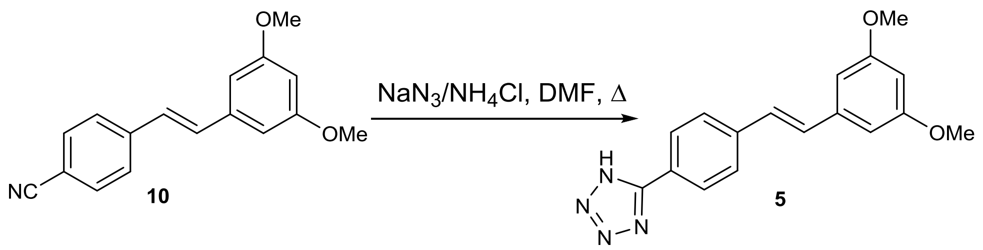

A solution of (*E*)-4-(3,5-dimethoxystyryl)benzonitrile **10** (1 g, 581.9 mmol, 1.00 equiv) in anhydrous DMF (6 ml) was placed in a flask that had been purged and maintained with an inert atmosphere of nitrogen. Ammonium chloride (0.302 g, 5.65 mmol, 1.5 equivalents) was added, followed by sodium azide (0.367 g, 5.65mmol, 1.5 equivalents). The resulting solution was stirred overnight at 100 °C, then allowed to cool to room temperature. The resulting precipitate was filtered off and washed with EtOAc (20 ml). The crude material was purified by column chromatography under gravity (10:90 MeOH:chloroform) to give tetrazole **5** as a white solid (420 mg, 37.1%).

mp = 214 ºC Rf (10:90 MeOH:chloroform) = 0.17 IR ν (cm^{-1}) = 3016, 2836, 2710, 1583, 1457, 1316, 1156, 1059, 971, 840, 680. ^{1}H NMR (400 MHz, $CDCl_3$) δ (ppm) = 3.75 (6H, s, -OMe), 6.43 (1H, t, *J* = 2 Hz, H4), 6.69 (2H, d, *J* = 2 Hz, H2 and H6), 7.13 (2H, s, H-C=C-H), 7.64 (2H, d, *J* = 8.4 Hz, H2’and H6’), 8.10 (2H, d, *J* 8.4 Hz, H3’and H5’), ^{13}C-NMR (100 MHz, $CDCl_3$) δ (ppm)= 55.4 (OMe), 100.4 (C4), 104.8 (C2, C6), 127.0 (C2’,C6’), 127.6

(C3',C5'), 127.4 (C4'), 128.0 (-C=C-), 130.5 (-C=C-), 138.8 (-C=N), 138.8 (C1), 139.7 (C1'), 161.0 (C3,C5). HRMS m/z calculated for $C_{17}H_{16}N_4NaO_2^+$ $[M+Na]^+$ requires 331.1171; 331.11242 $[M+Na]^+$ found 639.23572 $[2M+Na]^+$.

Synthesis (E)-5-(2-(3,5-dimethoxystyryl)phenyl)-1*H*-tetrazole (6)

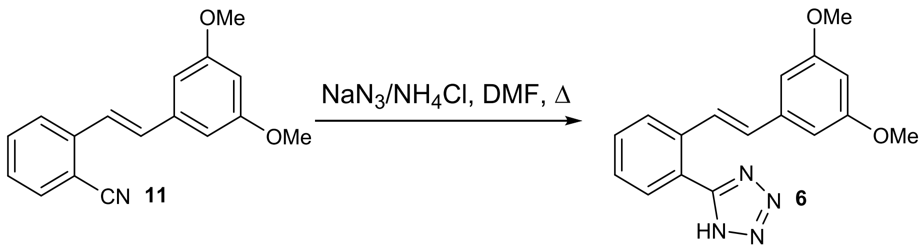

A solution of (*E*)-2-(3,5-dimethoxystyryl)benzonitrile **11** (1 g, 581.9 mmol, 1.00 equivalent) in anhydrous DMF (600 ml) was placed in a flask that had been purged and maintained with an inert atmosphere of nitrogen. Ammonium chloride (0.302 g, 5.65 mmol, 1.5 equivalents) was added, followed by sodium azide (0.367 g, 5.65 mmol, 1.5 equivalents). The resulting solution was stirred overnight at 100 °C, then allowed to cool to room temperature. The resulting precipitate was filtered off and washed with EtOAc (20 ml). The crude material was purified by column chromatography under gravity (10:90 MeOH:chloroform) to give the target tetrazole as a brown solid (190 mg, 16.4%).

mp = 214 ºC Rf (10:90 MeOH:chloroform) = 0.19. IR ν (cm^{-1}) = 2997, 2841, 2763, 1598, 1423, 1301, 1214, 1156, 1059, 961, 835, 753, 680. ^{1}H NMR (400 MHz, $CDCl_3$) δ (ppm) = 3.77 (6H, s, -OMe), 6.47 (1H, t, *J* = 2 Hz, H4), 6.70 (2H, d, *J* = 2 Hz, H2 and H6), 7.22 (1H, d, *J* = 16.2Hz, -C=C-H), 7.50 (1H, td, *J* = 7.6, 1.2 Hz, H5'), 7.61 (1H, td, *J* = 7.6, 1.2 Hz, H4'), 7.64 (1H, d, *J* = 16.2 Hz, -C=C-H), 7.75 (1H, dd, *J* = 1.2, 7.6 Hz, H6'), 7.97 (1H, d, *J* = 8 Hz, H3'). 13C NMR (100 MHz, $CDCl_3$) δ (ppm) = 55.4 (OMe), 99.8 (C4), 104.9 (C2, C6), 123.1 (C=N), 126.4 (-C=C-H, C3'), 128.0 (C5'), 129.9 (C6'), 130.9 (C4'), 131.3 (-C=C-), 136.2 (C1), 139.0 (C1'), 160.7 (C3, C5). HRMS m/z calculated for $C_{17}H_{16}N_4O_2Na$ requires 331.1171; found 331.11873 $[M + Na]^+$ and 281.13286 $[M-N_2+H]^+$.

Synthesis of (*E*)-4-(3,5-dimethoxystyryl)aniline (8)

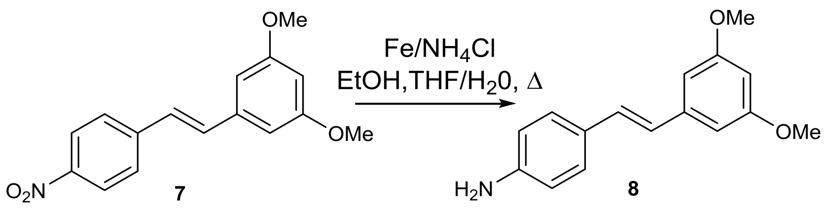

To a solution of (*E*)-1,3-dimethoxy-5-(4-nitrostyryl)benzene **7** (10 g, 35 mmol, 1 equivalent) in mixture of EtOH/THF/Water (100 ml in ratio 10:5:2.5) were added ammonium chloride (9.37g, 175.3 mmol, 5.0 equivalents) and iron powder (6.85 g, 122.6 mmol, 3.5 equiv) and the reaction mixture was heated to reflux at 80 ºC overnight. After completion of the reaction (by TLC), the reaction mixture was filtered through celite before cooling. This solution was evaporated to dryness under reduced pressure. The resulting crude was dissolved in saturated sodium bicarbonate solution (250 ml) and extracted with EtOAc (2 x100ml). The organic layer was washed with water (100 ml) and then with saturated brine (100 ml). This was then dried with excess anhydrous sodium sulphate and filtered under normal gravity. The filtrate was evaporated to dryness under reduced pressure. The crude product was purified by flash column chromatography (2:8 EtOAc:petroleum ether) to afford the aniline **8** (5.98 g, 67 %).

mp = 87-90 °C, (literature[1] mp = 90-91ºC), Rf (30:90 EtOAc:petroleum ether) = 0.3, IR ν (cm^{-1}) = 3409, 3307, 3001, 2831, 1593, 1515, 1204, 1151, 961, 825, 670. ^{1}H NMR (400 MHz, $CDCl_3$) δ (ppm) = 3.84 (6H, s, -OMe), 6.38 (1H, t, *J* = 2Hz, H4), 6.65 (2H, d, *J* =2.1 Hz, H2, H6), 6.69 (2H, dd, *J* = 6.8, 2 Hz, H2',H6'), 6.87 (1H, d, *J* = 16.4 Hz, -C=C-H), 7.02 (1H, d, *J* = 16.4 Hz, -C=C-H), 7.34 (2H, dd, *J* = 6.8, 2 Hz, H3', H5'). ^{13}C NMR (100 MHz, $CDCl_3$) δ (ppm) = 55.3 (OMe), 99.5 (C4), 104.3 (C2, C6), 115.2 (C2', C6'), 125.2 (C=C), 127.8 (C3', C5'), 127.9 (C1'), 129.3 (C=C), 140.1 (C1'), 146.3 (C4'), 161.0 (C3, C5).

Synthesis of (*E*)-N-(4-(3,5-dimethoxystyryl)phenyl)acetamide (9)

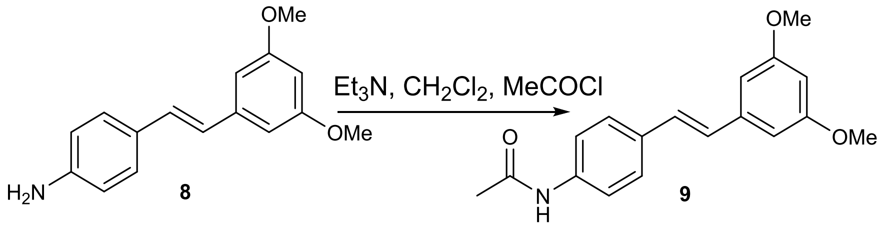

To a solution of aniline **8** (1 g, 3.91mmol, 1 equivalent) in dichloromethane (10 ml), triethylamine (3.1 g, 5.87 mmol, 1.5 equivalents) was added and then the mixture cooled to 0 °C with stirring. After 30 min, acetyl chloride (0.399 g, 5.09 mmol, 1.3 equivalents) was slowly added to the reaction mixture and this was then warmed to RT and stirred overnight. The resulting mixture was poured into water (25 ml) and extracted with EtOAc (2 x 25 ml). The combined organic layer was washed with 1N hydrochloric acid (1 x 5 ml) and water (1 x 10 ml). The organic layer was then evaporated under vacuum to give the crude product, and this was purified by column chromatography (EtOAc: petroleum ether 2:8) to obtain the acetamide **9** as a light brown solid (2.45 g, 70 %).

mp = 103°C, Rf (10:90 EtOAc:petroleum ether) = 0.22, IR ν (cm^{-1}) = 3337, 3079, 2924, 1666, 1583, 1204, 1156, 961, 821, 685. ^{1}H NMR (400 MHz, $CDCl_3$) δ (ppm) = 2.18 (3H, s, -COMe), 3.77 (6H, s, -OMe), 6.31 (1H, t, *J* = 2.1 Hz, H4), 6.59 (2H, d, *J* = 2.1 Hz, H2, H6), 6.88 (1H, d, *J* = 16.4 Hz, -C=C-H), 6.97 (1H, d, *J* = 16.4 Hz, -C=C-H), 7.36 (2H, d, *J* = 8.8 Hz, H2' and H6'), 7.65 (2H, d, *J* = 8.8Hz, H3', H5'), 9.12 (1H, s, -NH). ^{13}C NMR (100 MHz, $CDCl_3$) δ (ppm) = 24.4 (-CO<u>Me</u>), 55.3(OMe) 99.8 (C4), 104.5 (C2, C6), 120.0 (C3', C5'), 126.9 (C2', C6'), 127.3 (C=C), 128.7 (C=C), 132.4 (C1), 138.6 (C1'), 139.5 (C4'), 160.9 (C3, C5), 169.1 (CO). HRMS m/z calculated for $C_{18}H_{19}NNaO_3^+$ requires 320.1257 $[(M+Na)]^+$ found 320.11541 and 617.24234 $[(2M+Na)]^+$.

III. References for supplementary information

1. Saiyed, A.S. and Bedekar, A.V. One-pot synthesis of stilbenes by dehydrohalogenation–Heck olefination and multicomponent Wittig–Heck reaction. *Tetrahedron Letters* **51** 6227–6231 (2010)

IV. Copies of spectra

NMR and Mass Spectra of 3

^{1}H NMR spectrum of 3

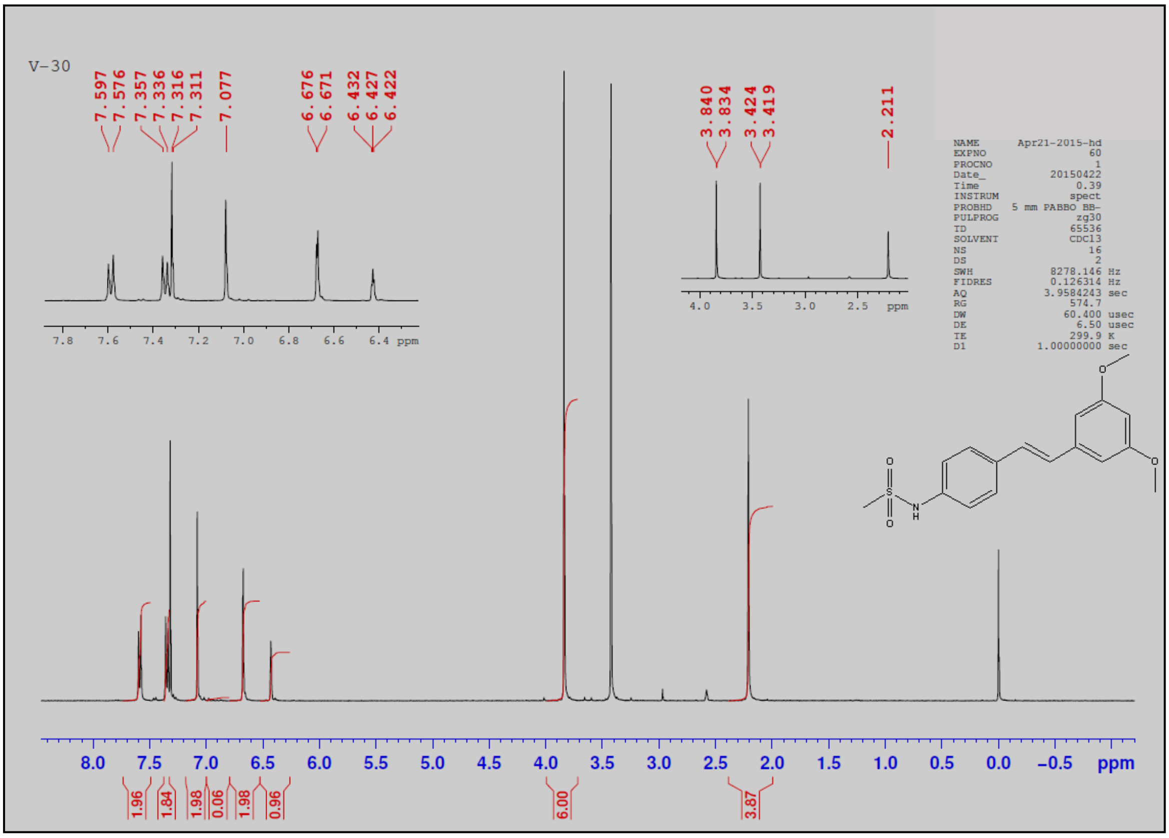

^{13}C NMR spectrum of 3

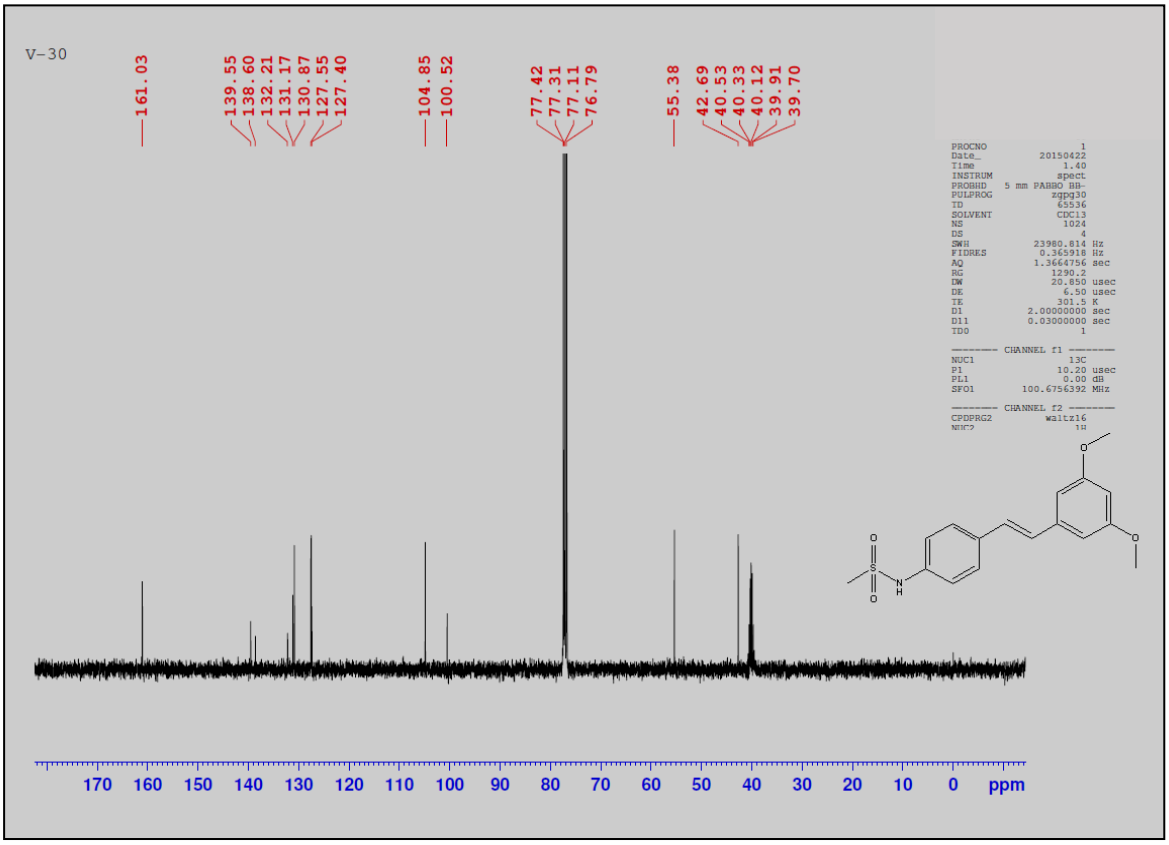

2D HSQC spectrum of 3

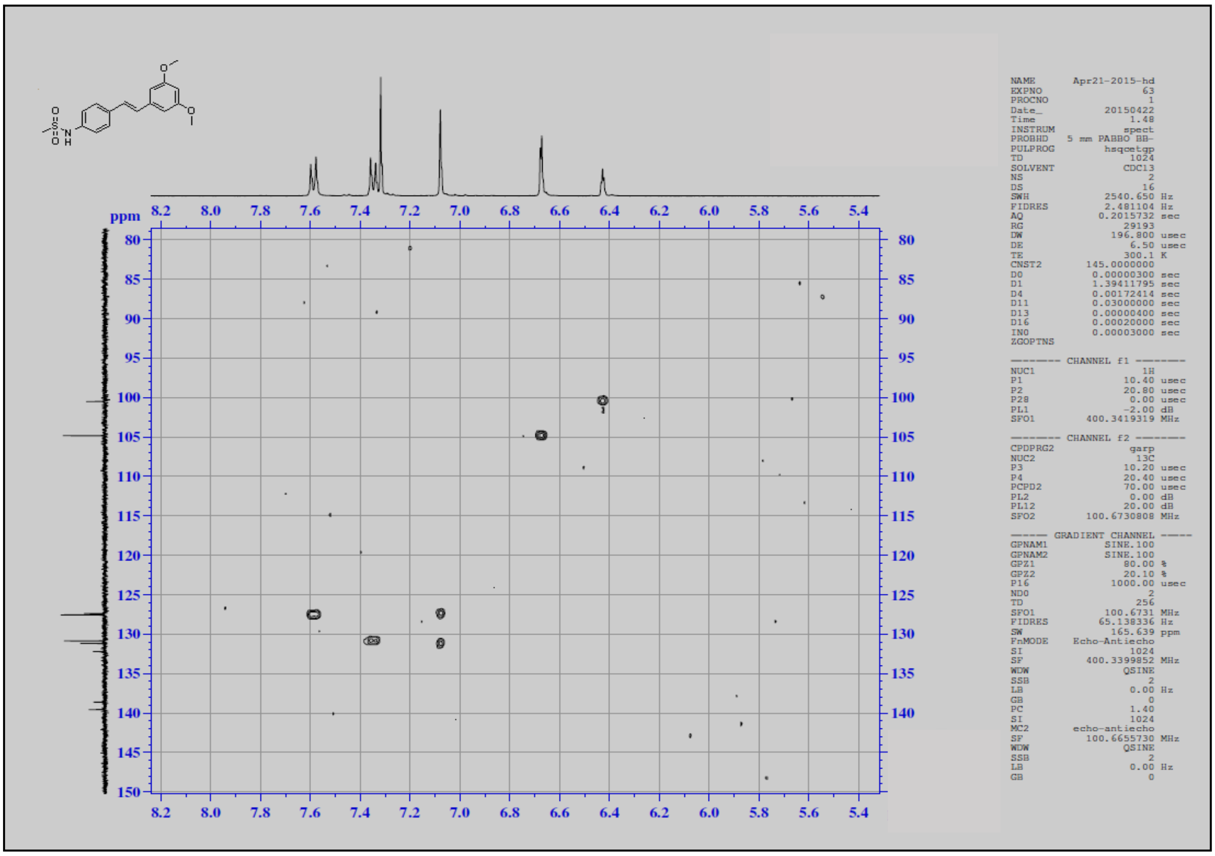

2D COSY spectrum of 3

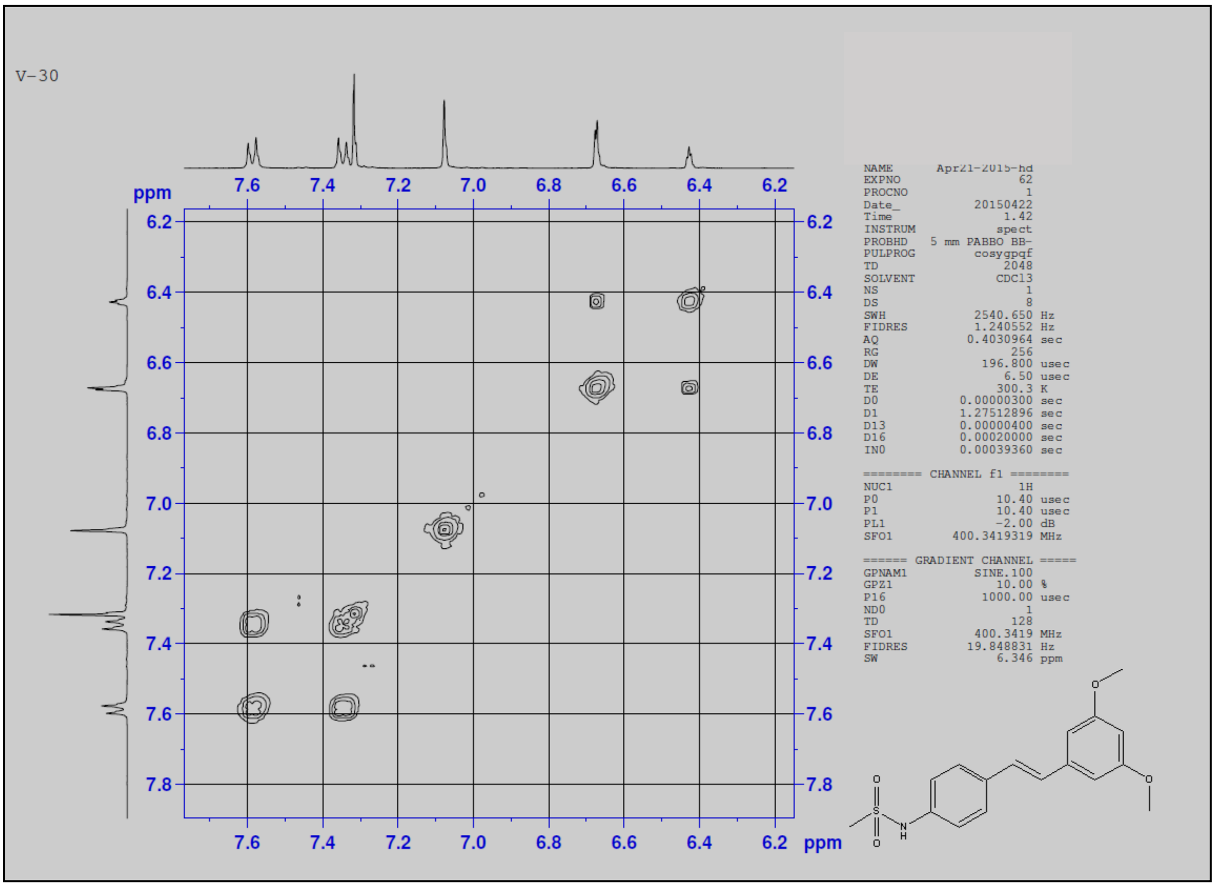

Mass spectrum of 3

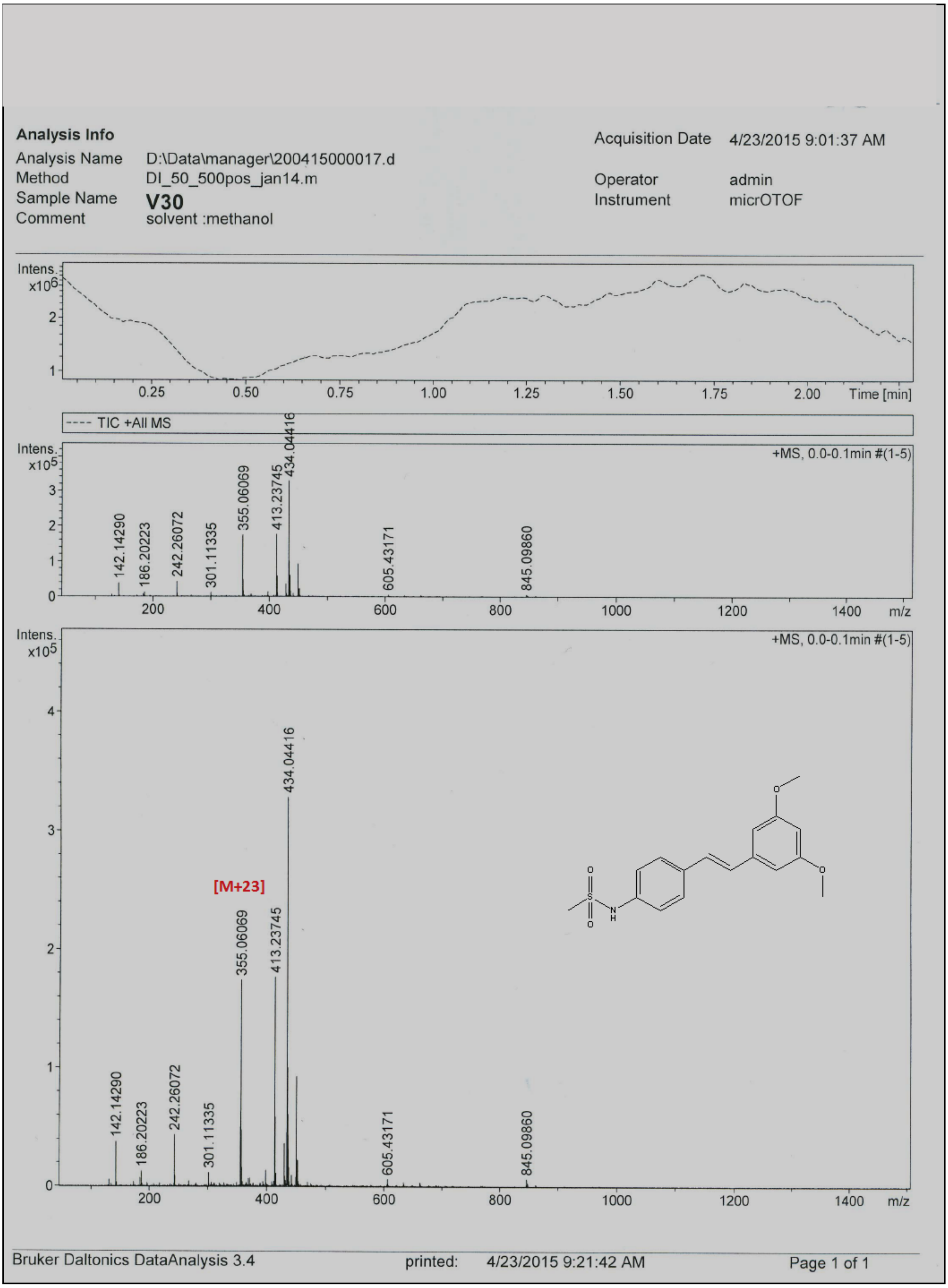

NMR and Mass Spectra of 4

^{1}H NMR spectrum of 4

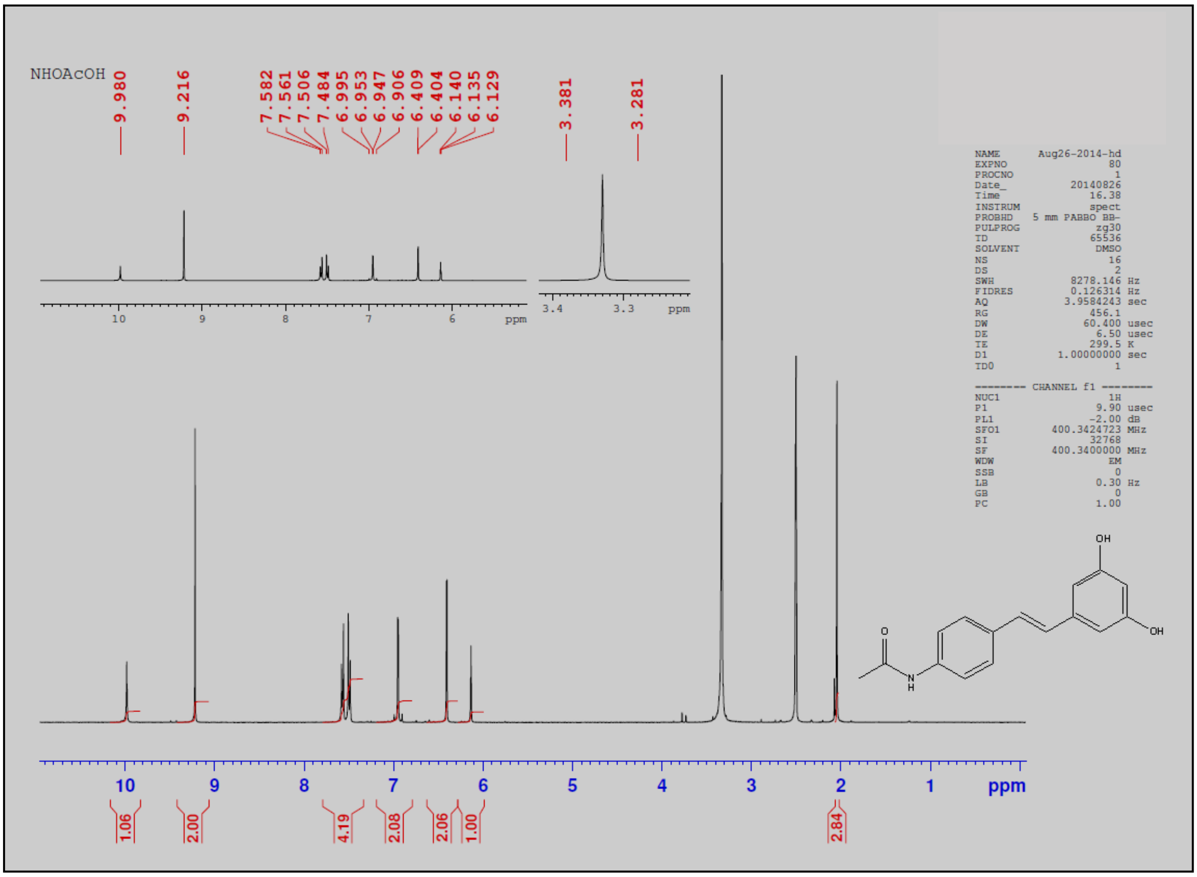

^{13}C NMR spectrum of 4

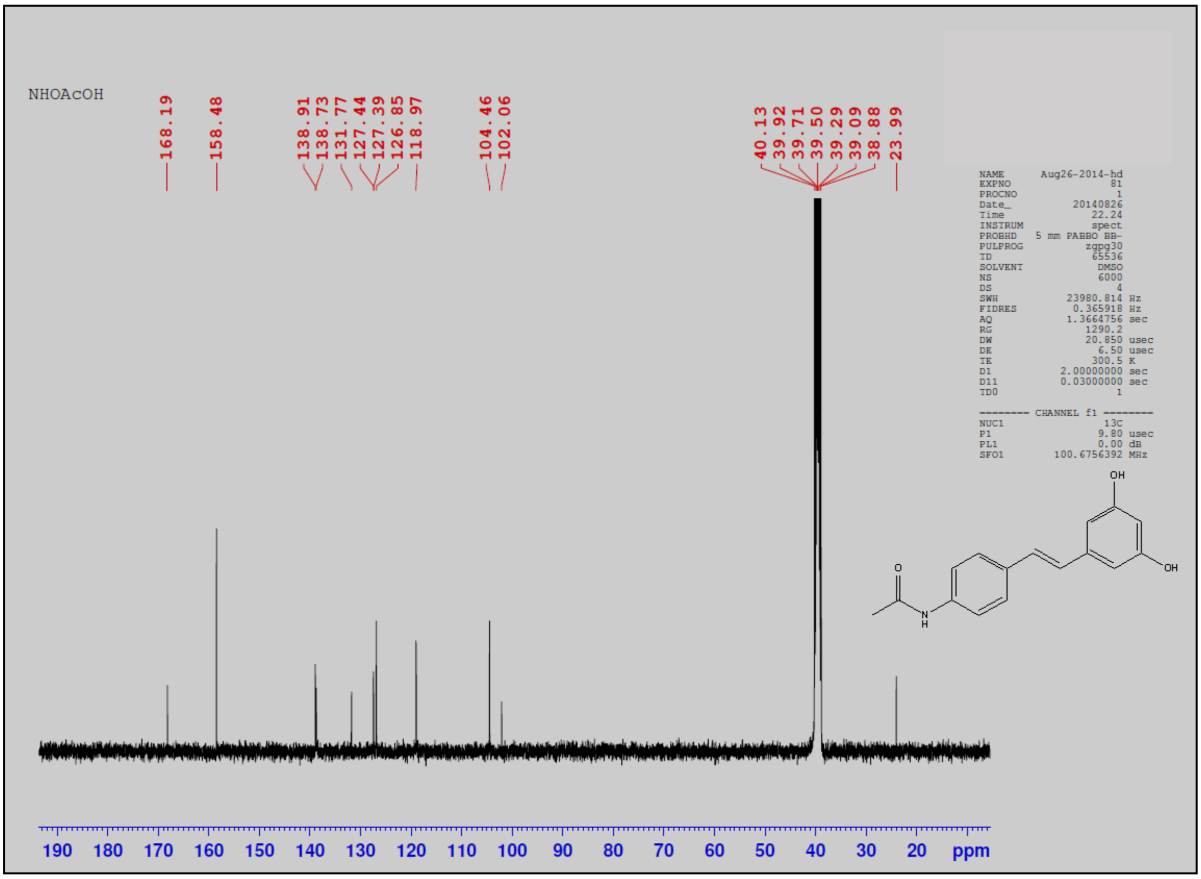

2D HSQC spectrum of 4

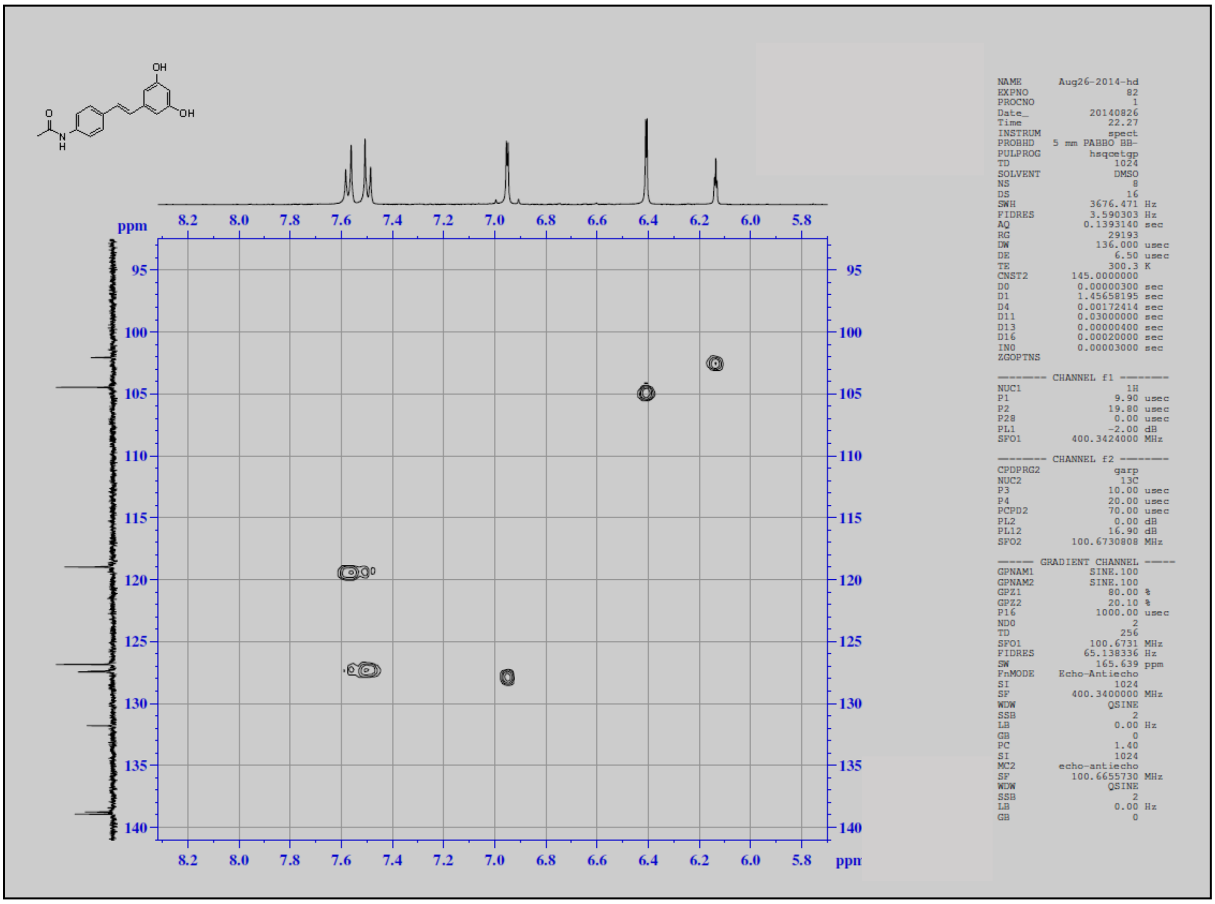

Mass spectrum of 4

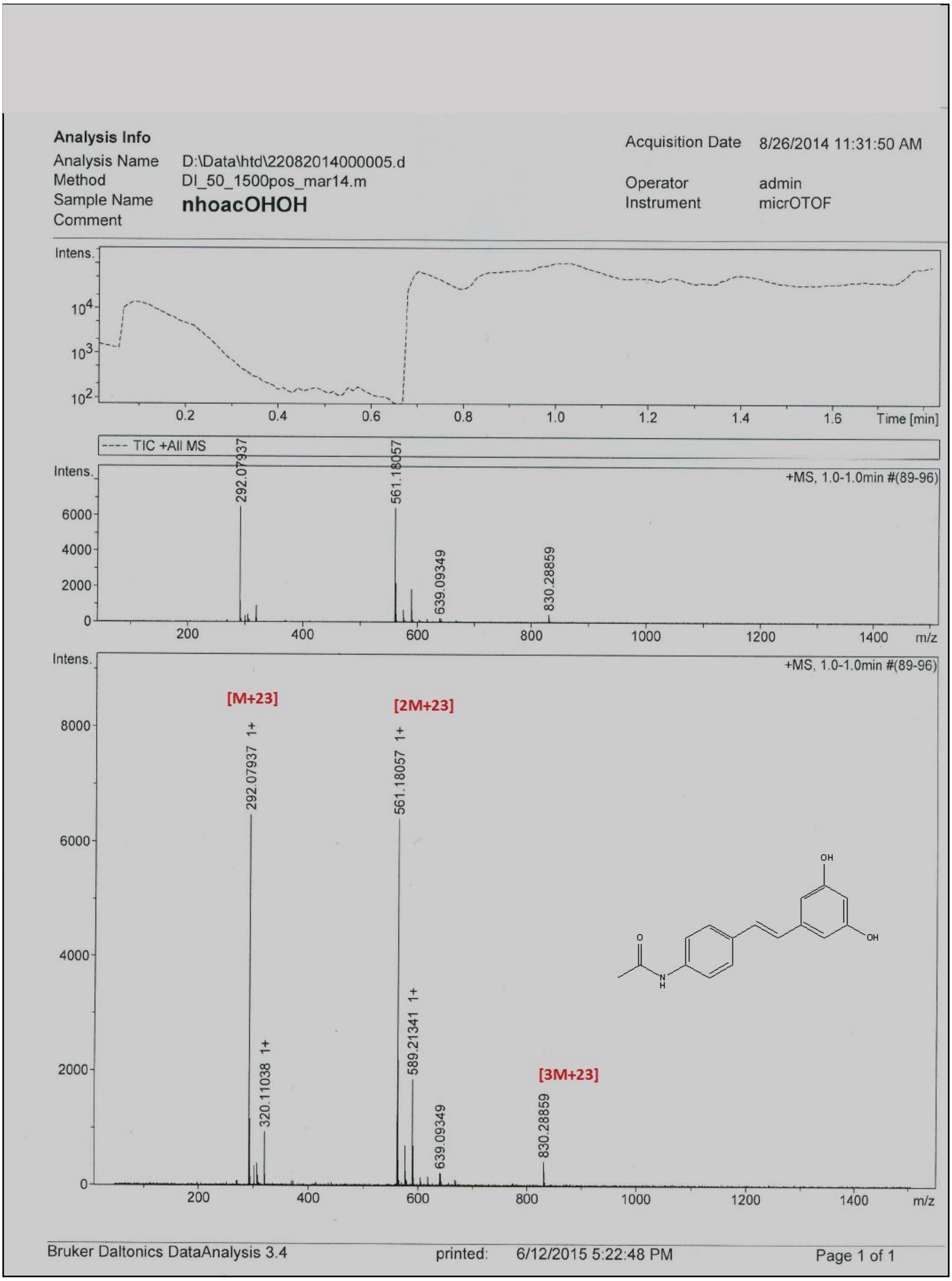

NMR and Mass Spectra of 5

^{1}H NMR spectrum of 5

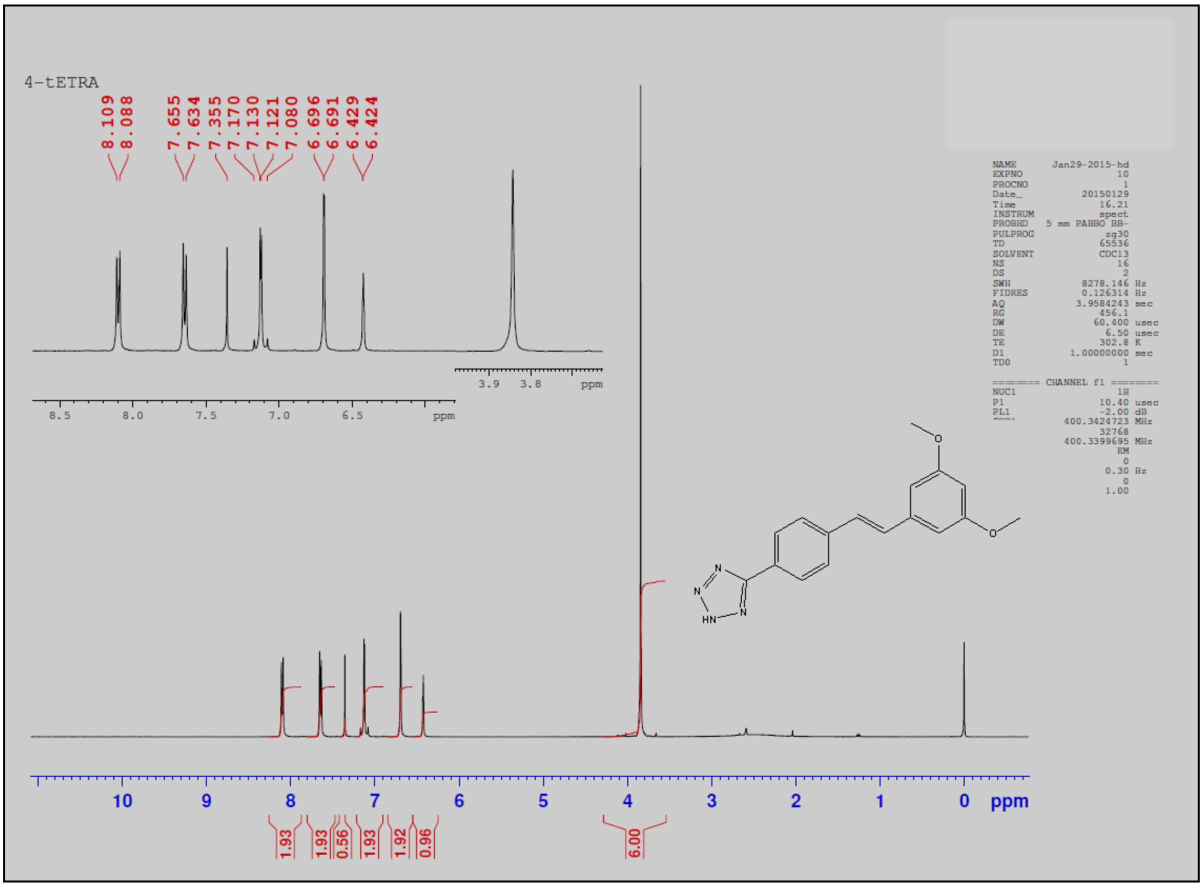

^{13}C NMR spectrum of 5

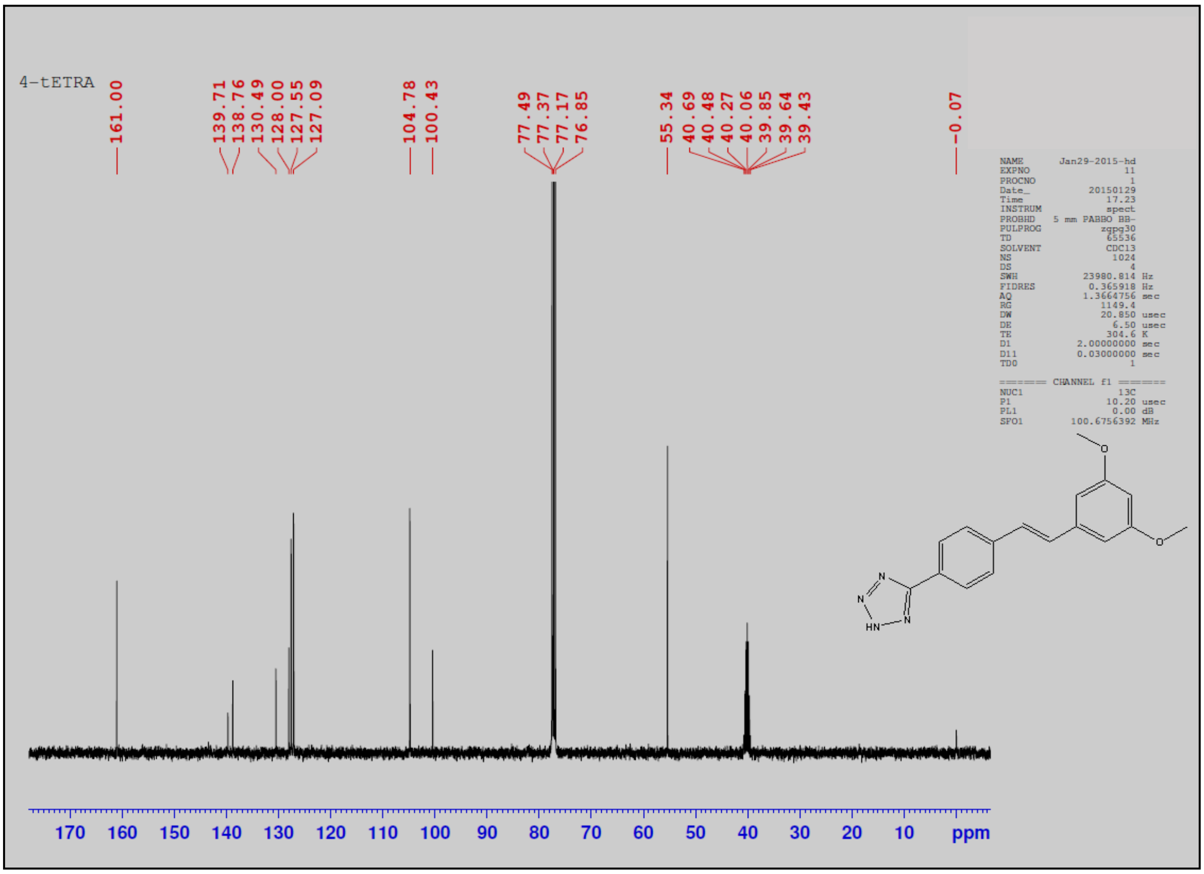

2D HSQC spectrum of 5

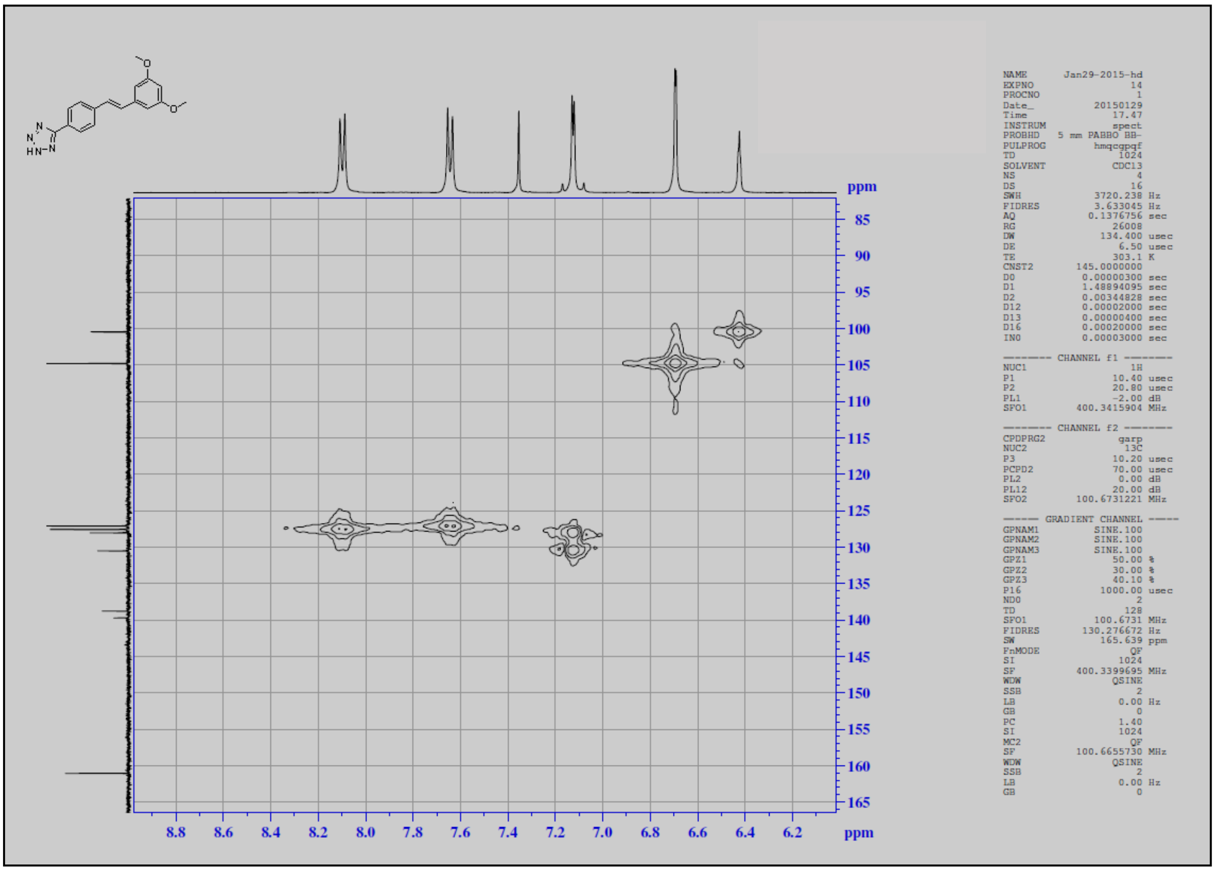

2D COSY spectrum of 5

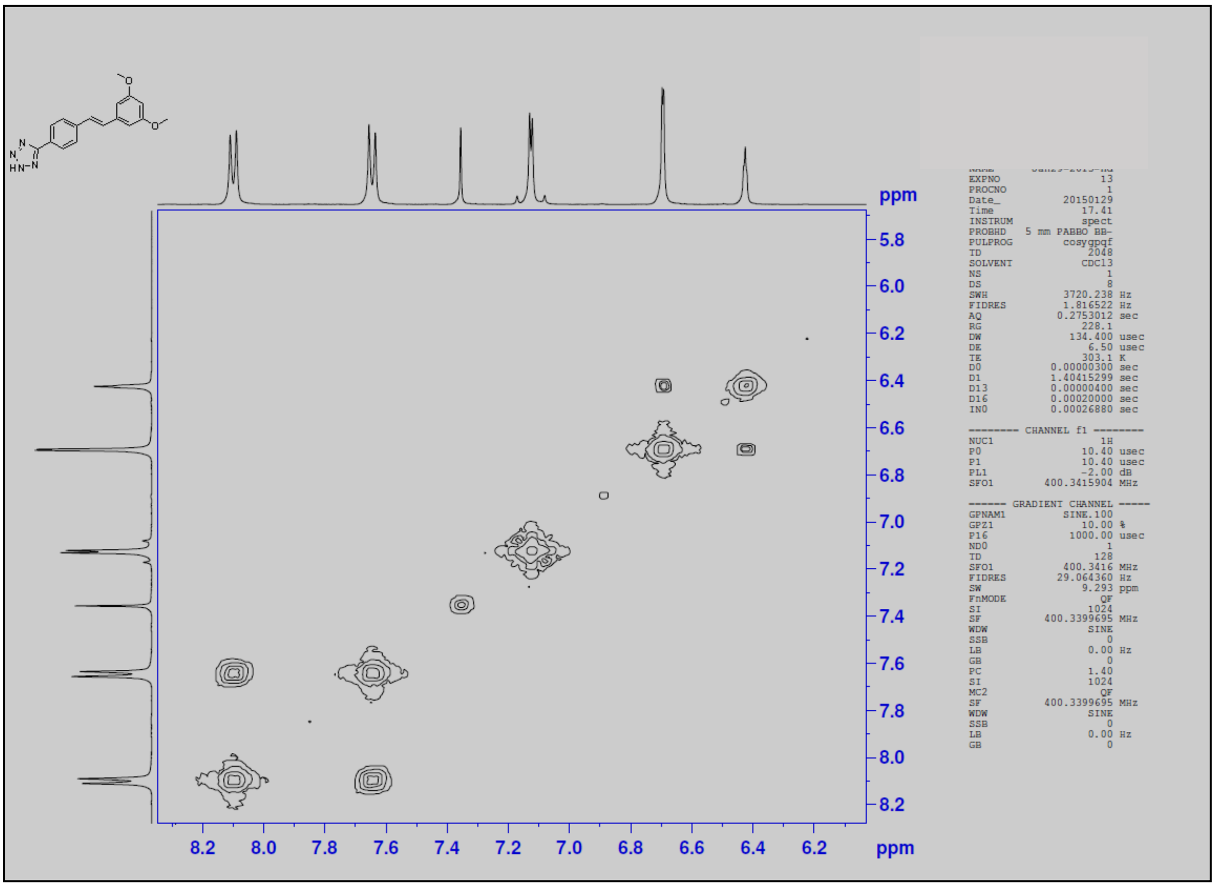

Mass spectrum of 5

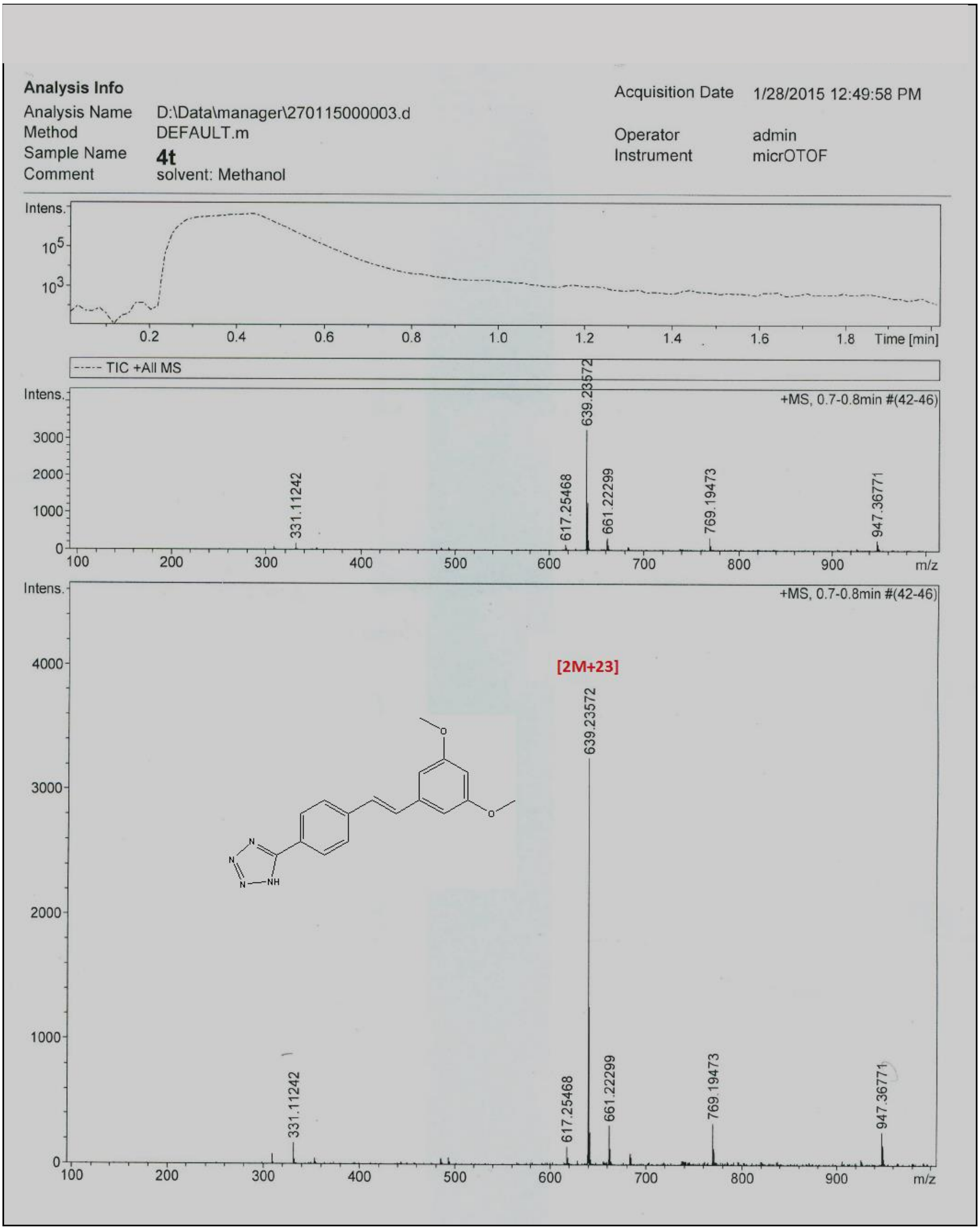

NMR and Mass Spectra of 6

^{1}H NMR spectrum of 6

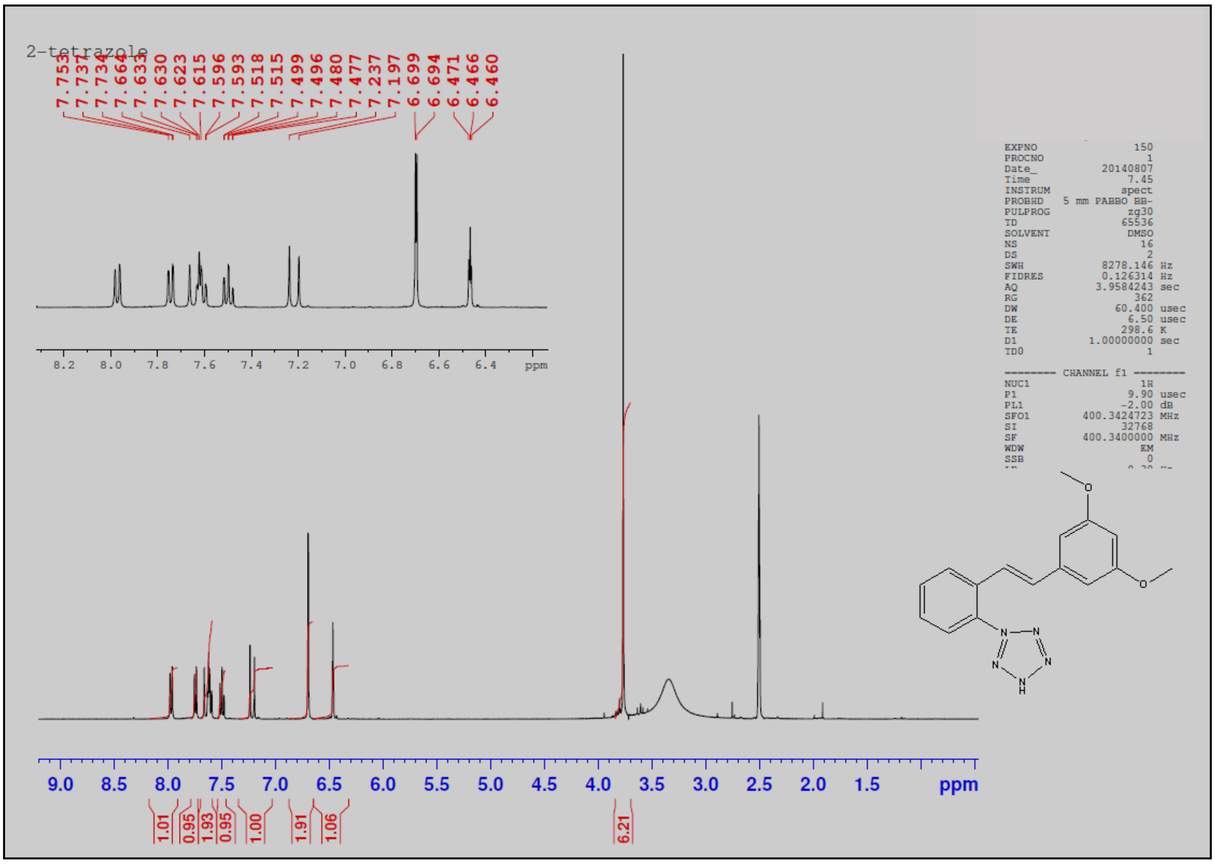

^{13}C NMR spectrum of 6

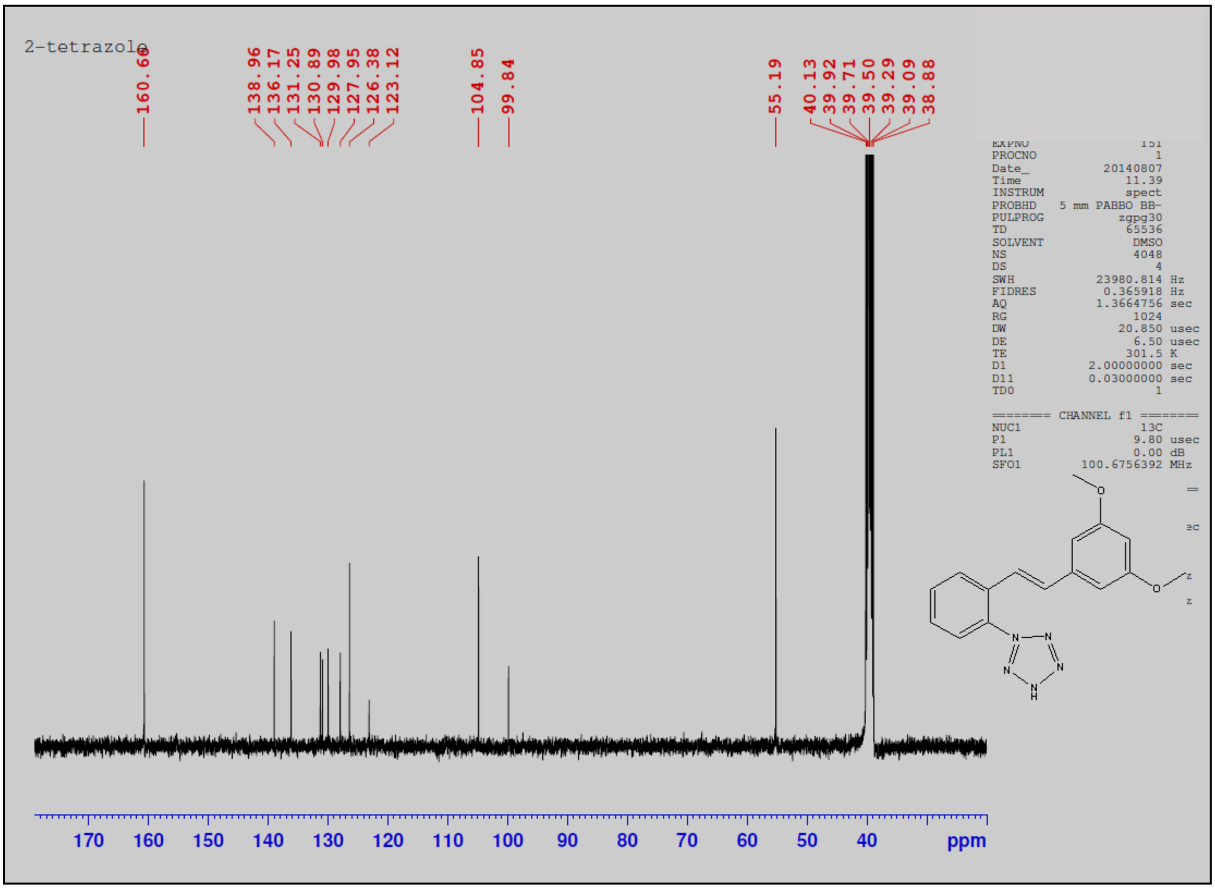

2D HSQC spectrum of 6

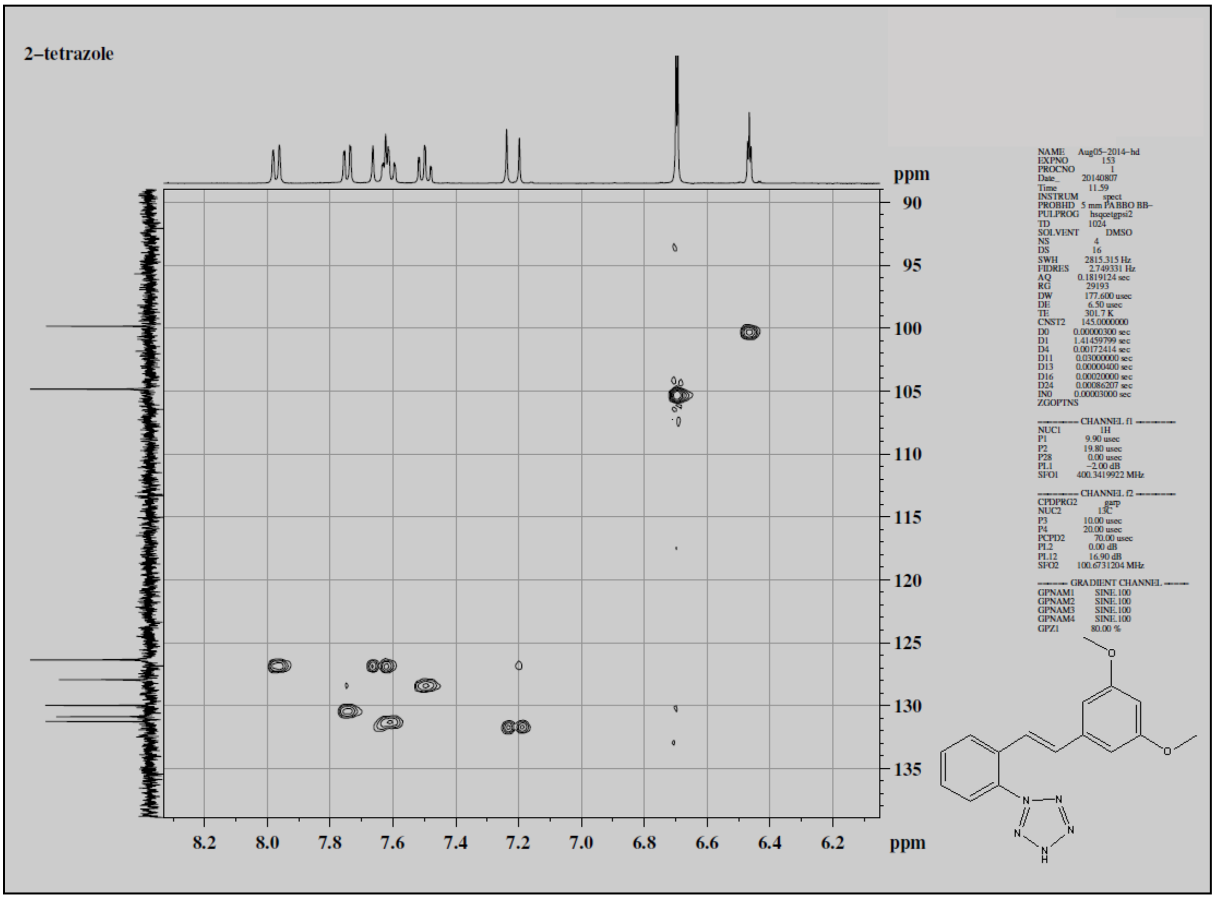

2D COSY spectrum of 6

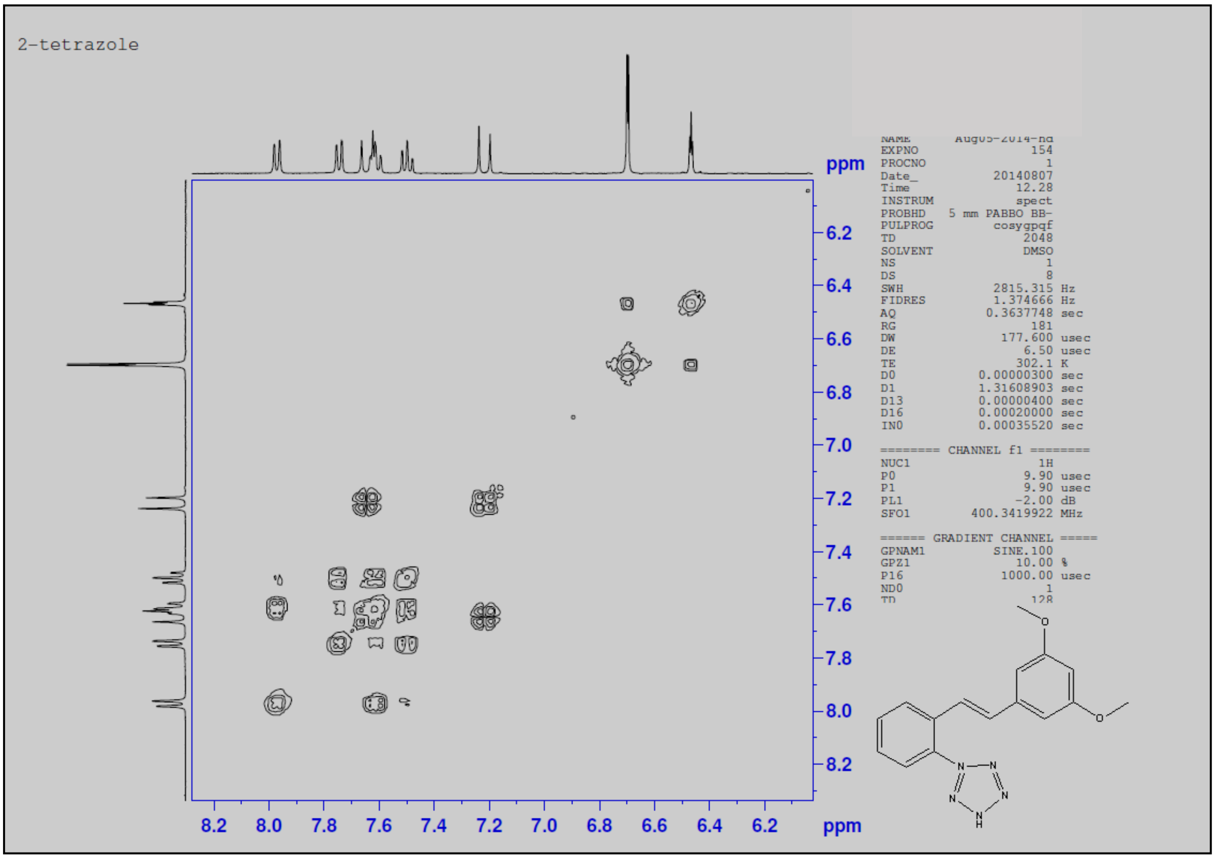

Mass spectrum of 6

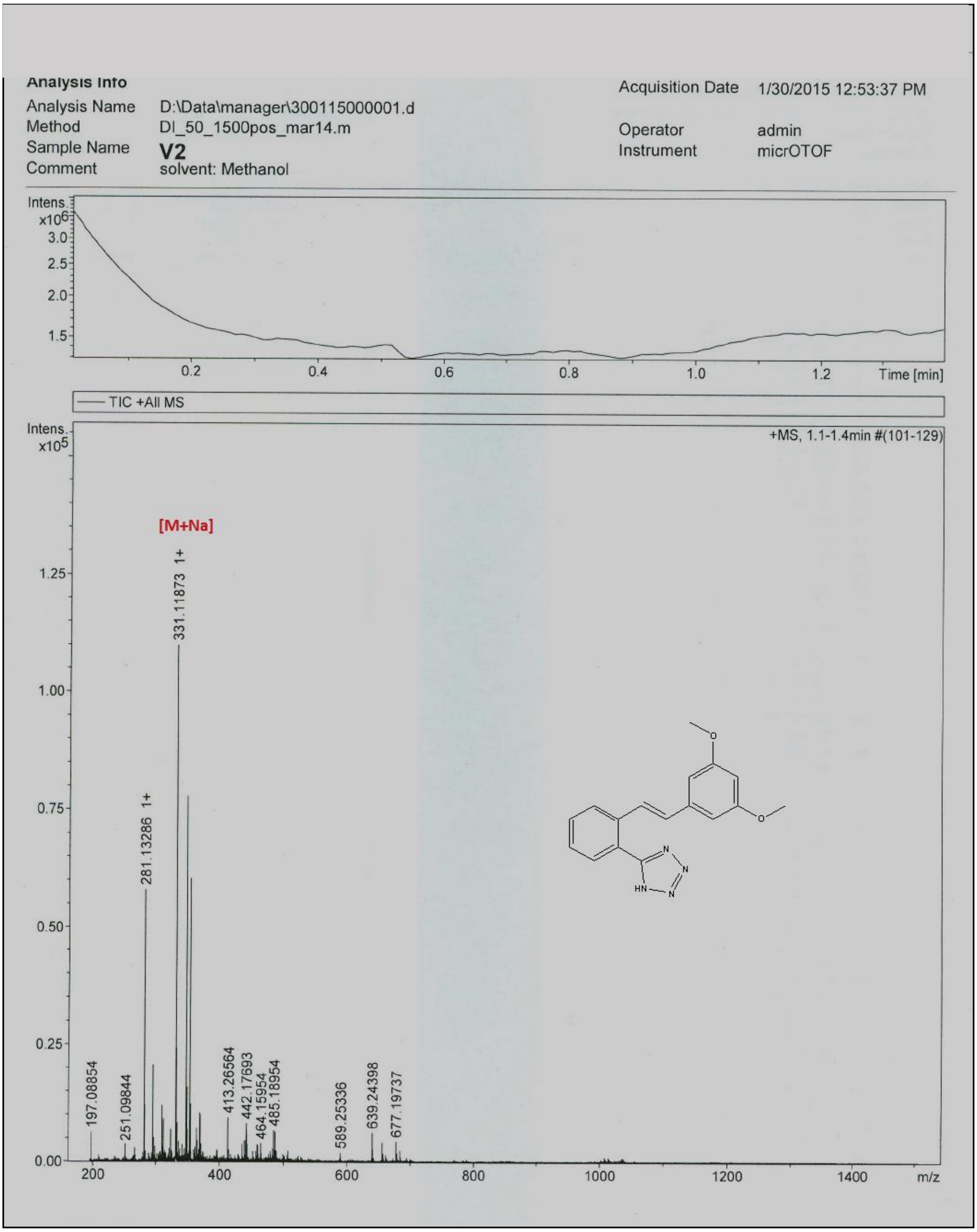

NMR and Mass Spectra of 8

^{1}H NMR spectrum of 8

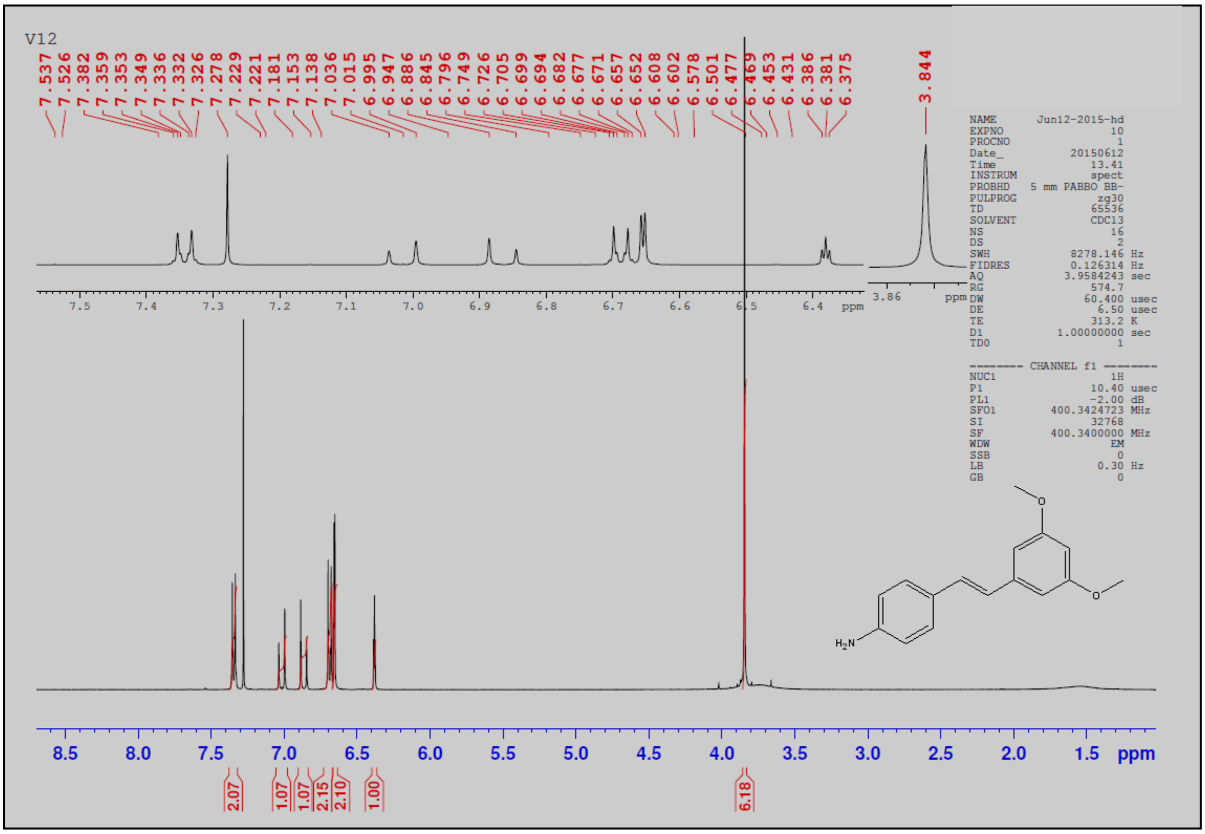

^{13}C NMR spectrum of 8

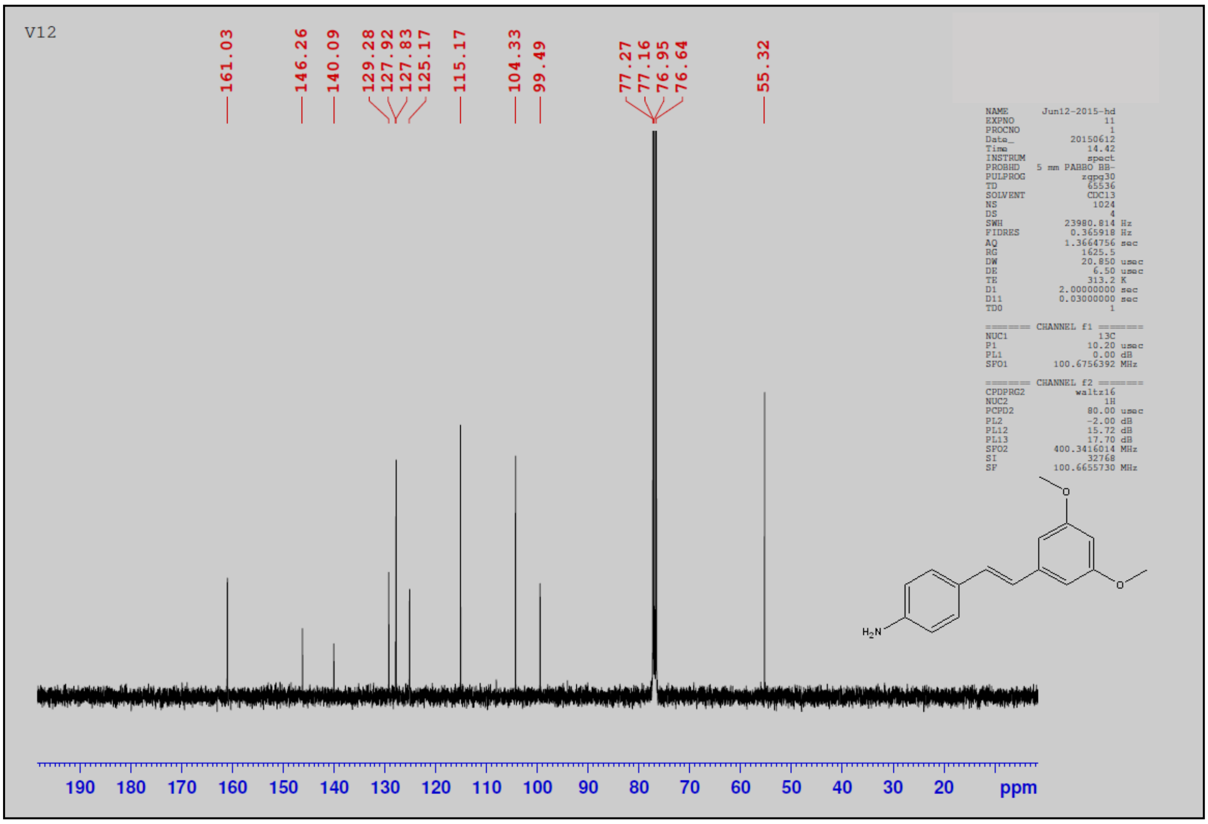

2D HSQC spectrum of 8

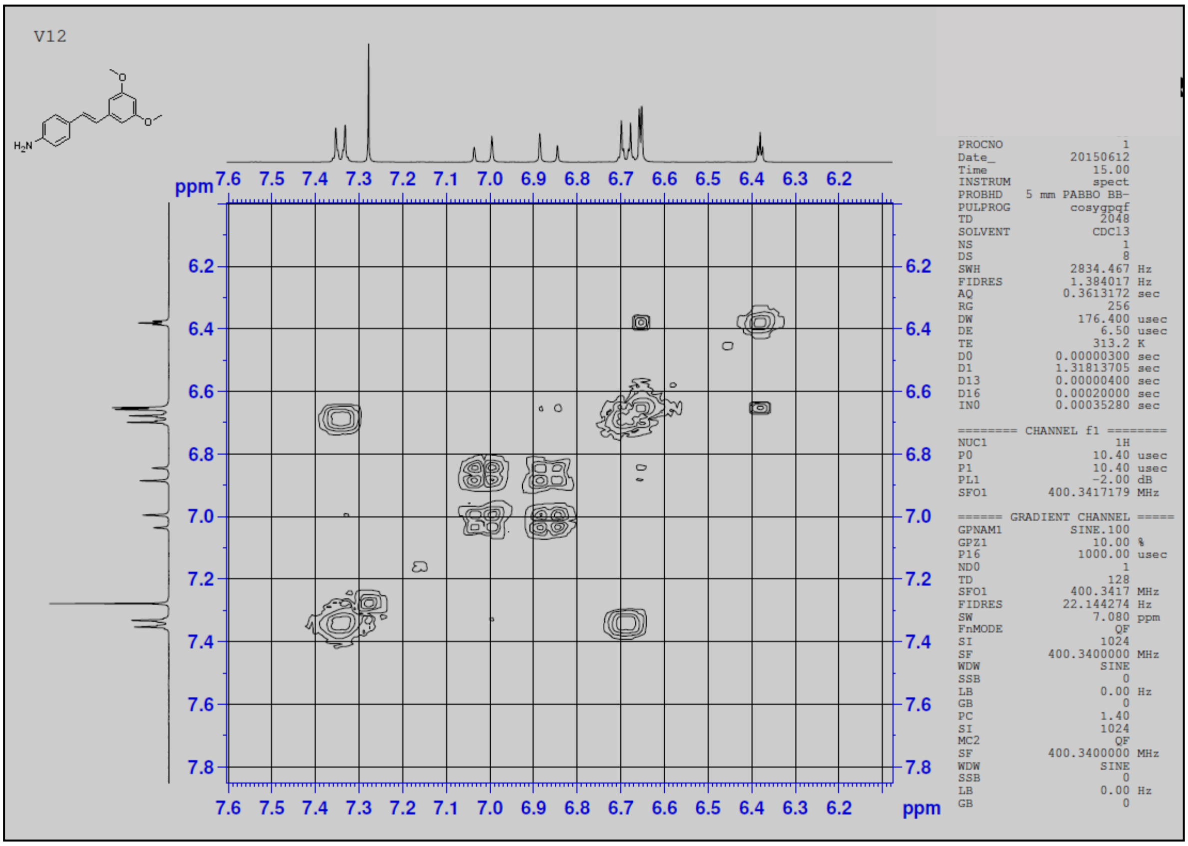

2D COSY spectrum of 8

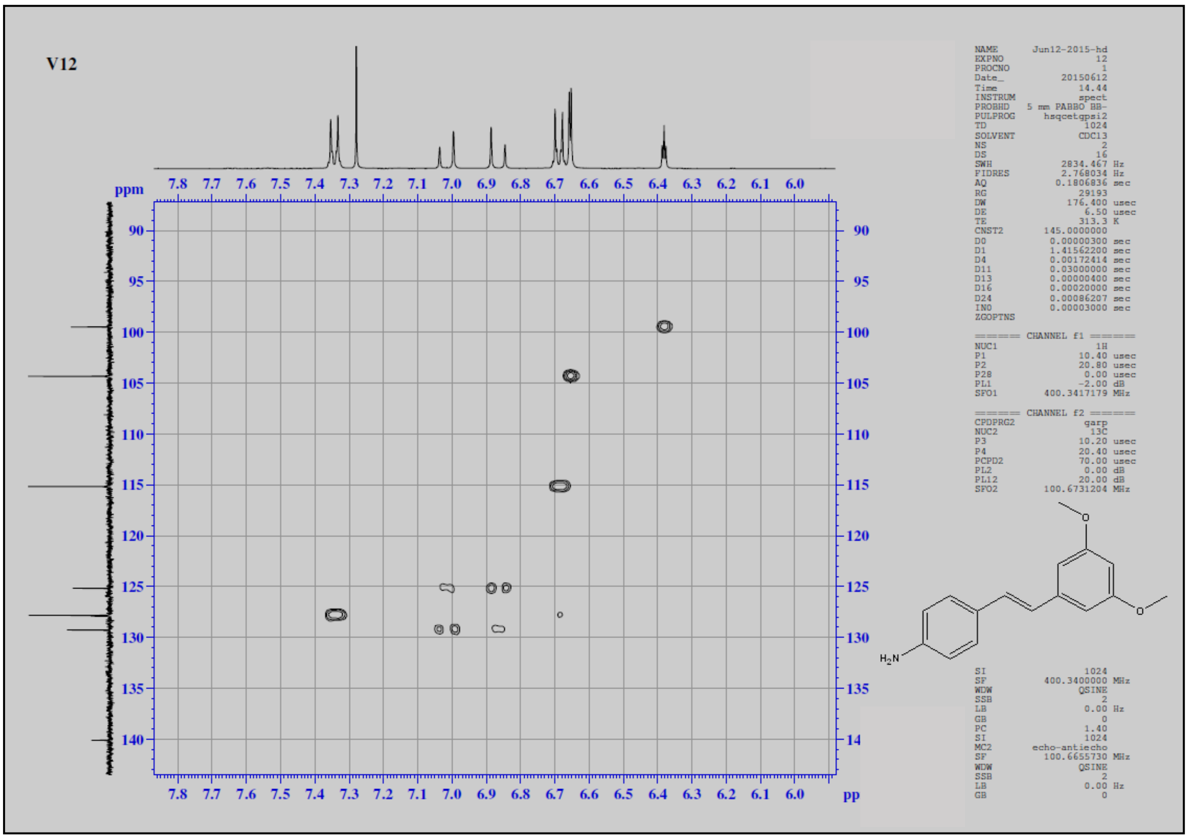

NMR and Mass Spectra of 9

^{1}H NMR spectrum of 9

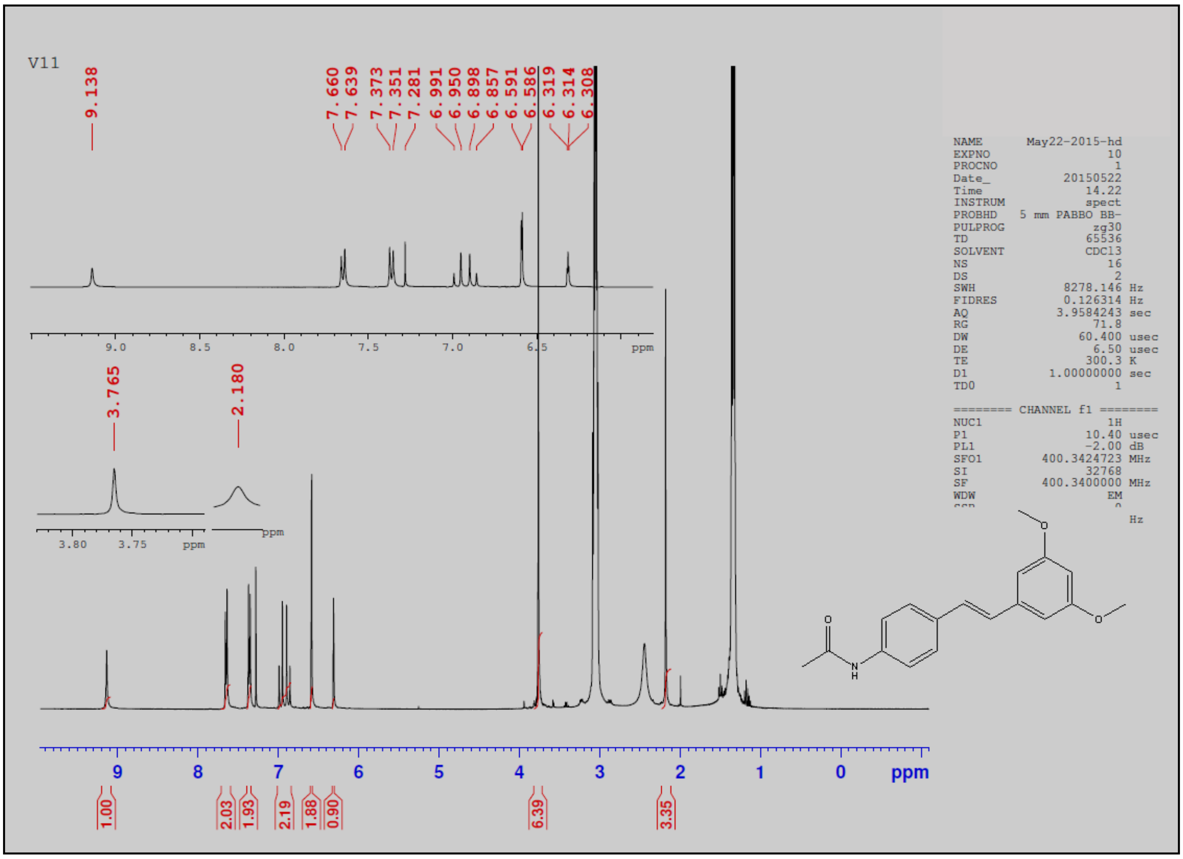

^{13}C NMR spectrum of 9

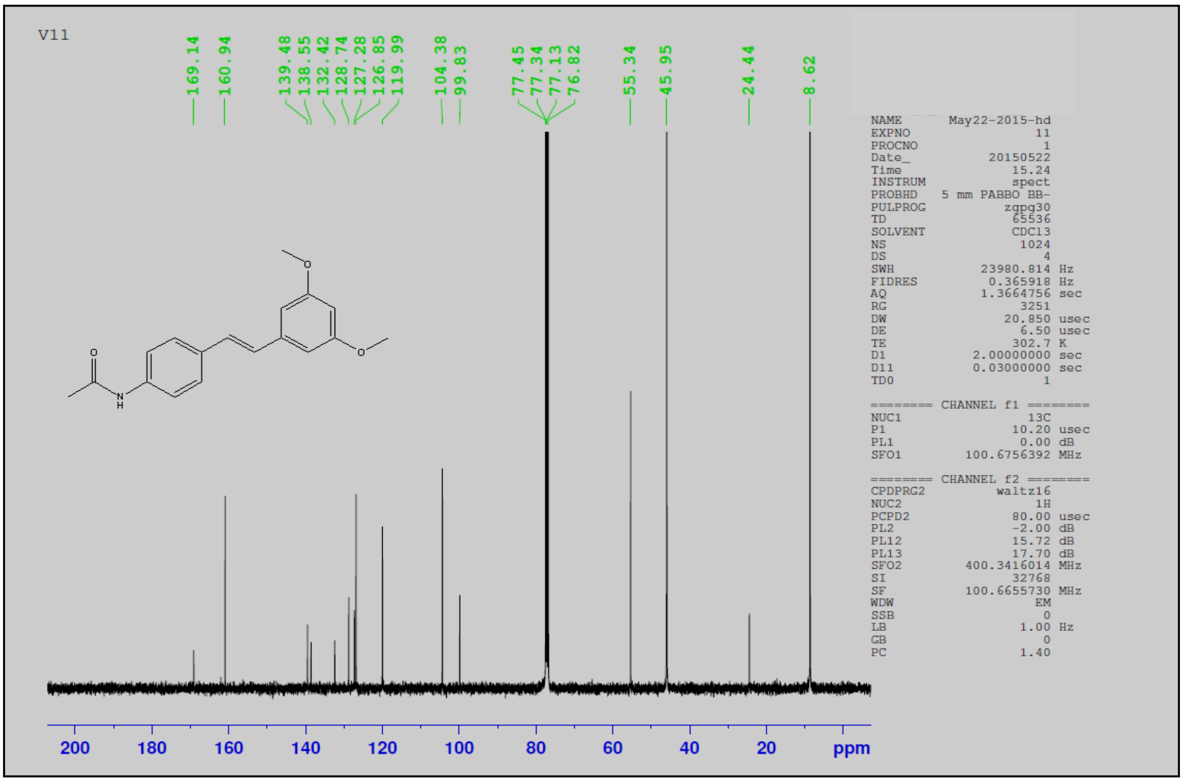

2D HSQC spectrum of 9

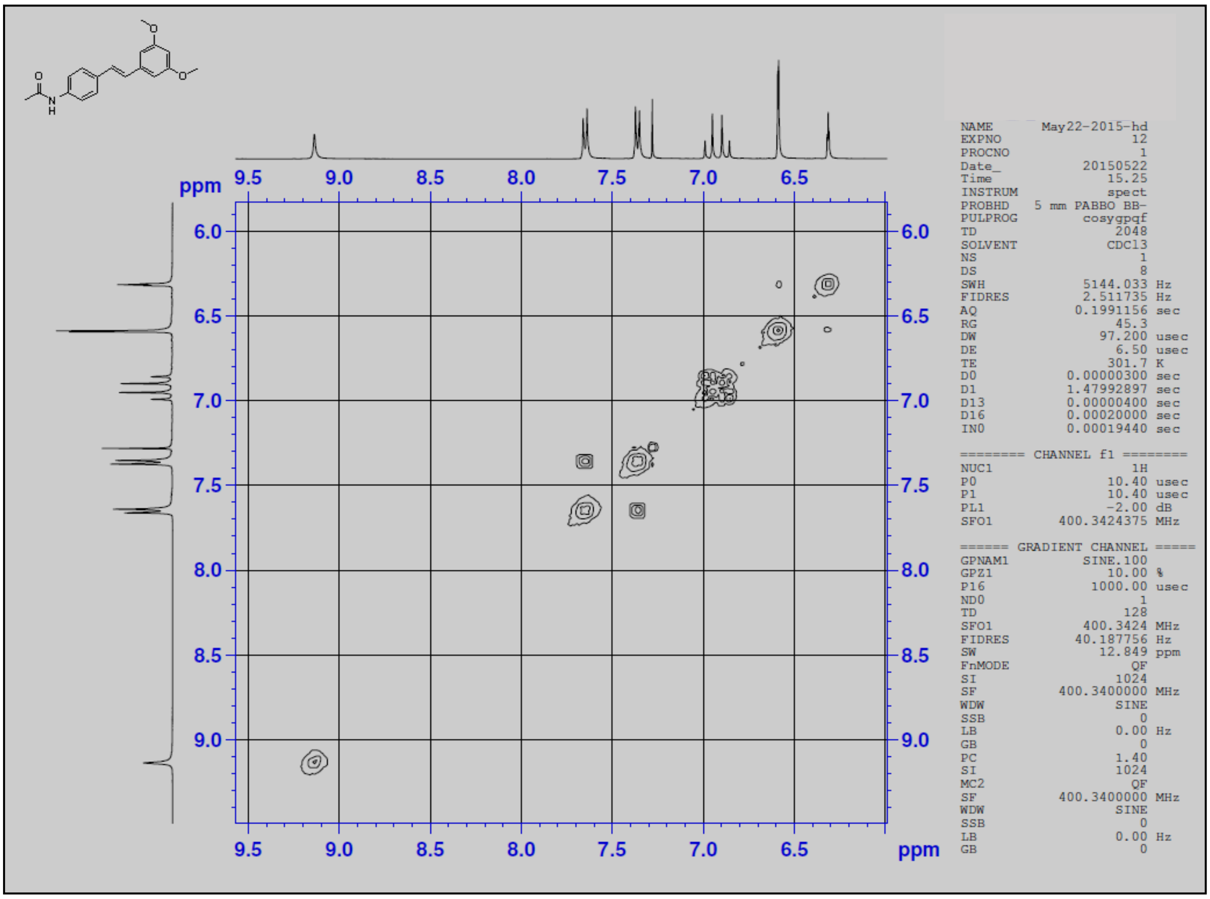

2D COSY spectrum of 9

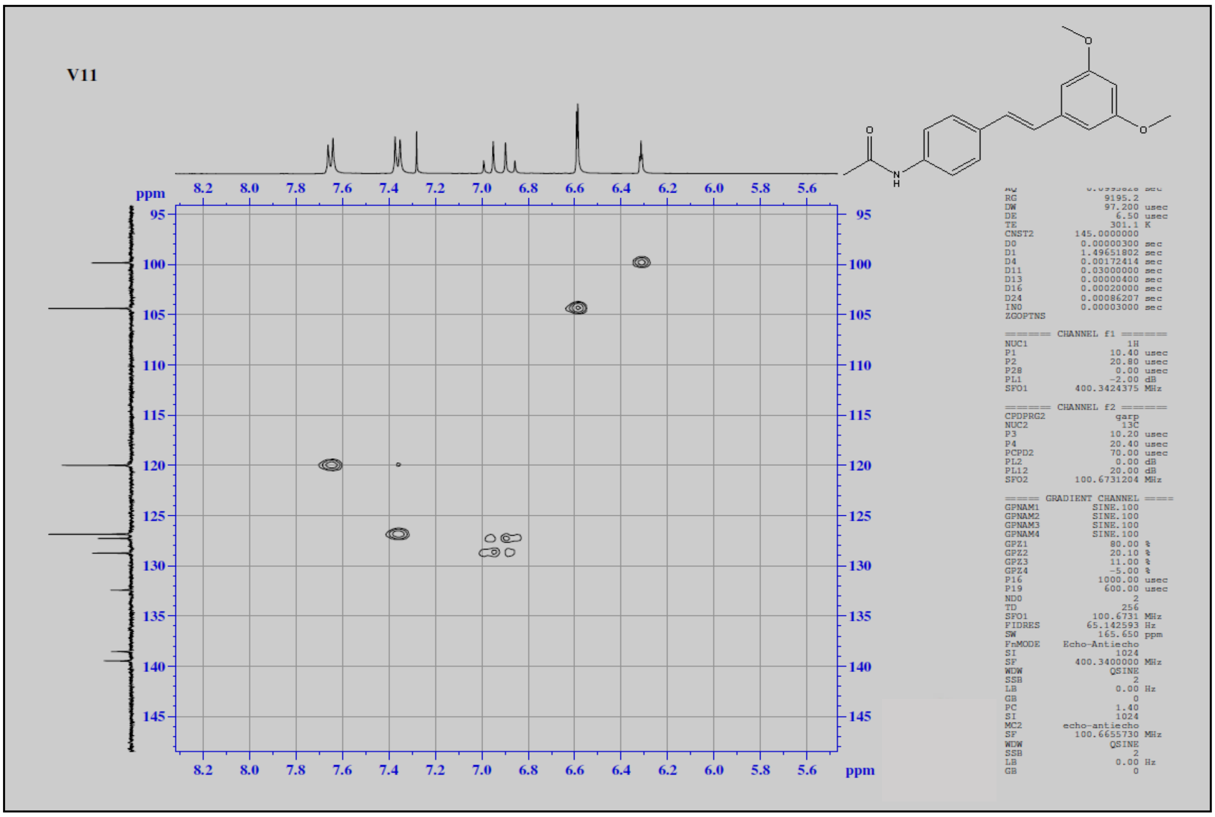

Mass spectrum of 9

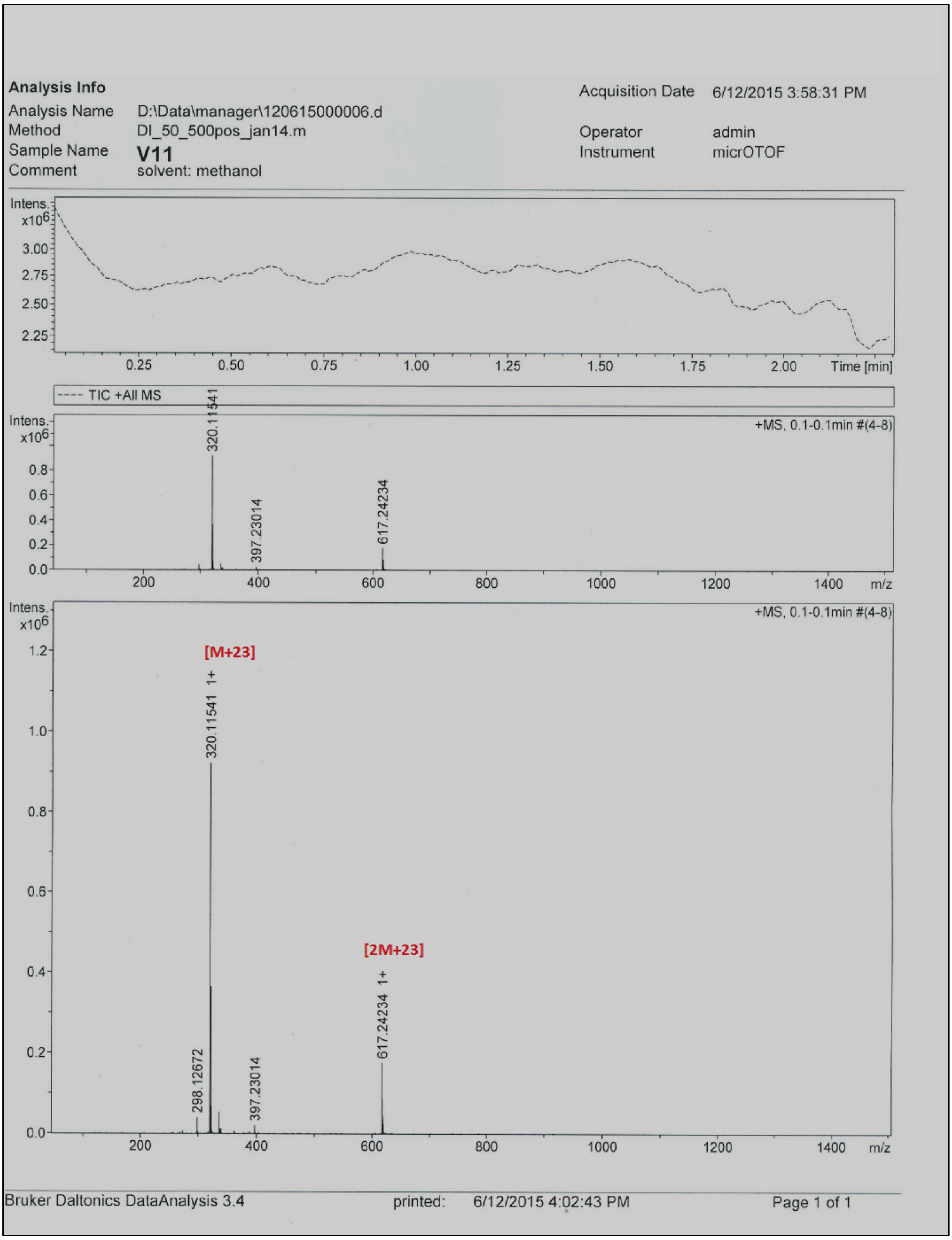

Supplement: Supplementary file 8 — Synthesis and characterisation of resveralogues. (PDF 3019 kb) [file 12860_2017_147_MOESM8_ESM.pdf]
